# Rapha Myr^®^, a Blend of Sulforaphane and Myrosinase, Exerts Antitumor and Anoikis-Sensitizing Effects on Human Astrocytoma Cells Modulating Sirtuins and DNA Methylation

**DOI:** 10.3390/ijms21155328

**Published:** 2020-07-27

**Authors:** Barbara Tomasello, Maria Domenica Di Mauro, Giuseppe Antonio Malfa, Rosaria Acquaviva, Fulvia Sinatra, Giorgia Spampinato, Samuele Laudani, Giusy Villaggio, Anna Bielak-Zmijewska, Wioleta Grabowska, Ignazio Alberto Barbagallo, Maria Teresa Liuzzo, Elisabetta Sbisà, Maria Grazia Forte, Claudia Di Giacomo, Massimo Bonucci, Marcella Renis

**Affiliations:** 1 Department of Drug Science, Section of Biochemistry, University of Catania, Viale A. Doria 6, 95125 Catania, Italy; mariadomenica.dimauro@virgilio.it (M.D.D.M.); g.malfa@unict.it (G.A.M.); racquavi@unict.it (R.A.); ignazio.barbagallo@unict.it (I.A.B.); cdigiac@unict.it (C.D.G.); 2 Department of Biomedical and Biotechnological Sciences, University of Catania, Via Santa Sofia 87, 95125 Catania, Italy; sinatra@unict.it (F.S.); s.laudani91@hotmail.it (S.L.); giusyvillaggio@gmail.com (G.V.); 3 Services Center B.R.I.T. of the University of Catania, 95124 Catania, Italy; giorgiaspampinato@unict.it; 4 Nencki Institute of Experimental Biology, Polish Academy of Sciences, 3 Pasteur St, 02-093 Warsaw, Poland; a.bielak@nencki.gov.pl (A.B.-Z.); w.grabowska@nencki.gov.pl (W.G.); 5 Medical Clinic of Clinical Allergology and Immunology, 97018 Scicli, Italy; mariateresaliuzzo@gmail.com; 6 Institute of Biomedical Technologies -National Research Council Bari, 70126 Bari, Italy; elisabetta.sbisa@ba.itb.cnr.it; 7 Dept. Prevention ASL BA—SIAN Metropolitan Area Bari, 70120 Bari, Italy; mariagrazia.forte@asl.bari.it; 8 Association Research Center for Integrative Oncology Treatments (ARTOI), 00165 Rome, Italy; maxbonucci@artoi.it

**Keywords:** brain cancer, astrocytoma 1321N1 cells, sulforaphane, myrosinase, cytoskeleton morphology, cell migration, anoikis, apoptosis, oxidative stress, global DNA methylation, sirtuins, mutated p53 R213Q

## Abstract

Brain and other nervous system cancers are the 10th leading cause of death worldwide. Genome instability, cell cycle deregulation, epigenetic mechanisms, cytoarchitecture disassembly, redox homeostasis as well as apoptosis are involved in carcinogenesis. A diet rich in fruits and vegetables is inversely related with the risk of developing cancer. Several studies report that cruciferous vegetables exhibited antiproliferative effects due to the multi-pharmacological functions of their secondary metabolites such as isothiocyanate sulforaphane deriving from the enzymatic hydrolysis of glucosinolates. We treated human astrocytoma 1321N1 cells for 24 h with different concentrations (0.5, 1.25 and 2.5% *v/v*) of sulforaphane plus active myrosinase (Rapha Myr^®^) aqueous extract (10 mg/mL). Cell viability, DNA fragmentation, PARP-1 and γH2AX expression were examined to evaluate genotoxic effects of the treatment. Cell cycle progression, p53 and p21 expression, apoptosis, cytoskeleton morphology and cell migration were also investigated. In addition, global DNA methylation, DNMT1 mRNA levels and nuclear/mitochondrial sirtuins were studied as epigenetic biomarkers. Rapha Myr^®^ exhibited low antioxidant capability and exerted antiproliferative and genotoxic effects on 1321N1 cells by blocking the cell cycle, disarranging cytoskeleton structure and focal adhesions, decreasing the integrin α5 expression, renewing anoikis and modulating some important epigenetic pathways independently of the cellular p53 status. In addition, Rapha Myr^®^ suppresses the expression of the oncogenic p53 mutant protein. These findings promote Rapha Myr^®^ as a promising chemotherapeutic agent for integrated cancer therapy of human astrocytoma.

## 1. Introduction

Currently, the strategies involved in the prevention, progression, remission and relapse of cancer represent the key priority aims in public health worldwide [1,2].

Anaplastic astrocytomas and glioblastomas (GBM) represent the most frequent and aggressive primary malignancies of the central nervous system. So far, they are incurable diseases due mostly to their highly invasive phenotype, and the poor efficiency of the available therapies further reduced by the blood brain barrier that limits the delivery of drugs into the brain. Moreover, the current chemotherapy may lead to drug resistance in GBM treatment. Therefore, the treatment of these tumors constitutes a fundamental challenge for neuro-oncology and there is an urgent need to fully understand the molecular background of these cancers and to develop new promising therapeutic strategies [3].

The hallmarks of tumorigenesis and cancer progression are genetic instability, deregulation of epigenetic mechanisms, oxidative homeostasis, cell cycle progression, tumor microenvironment, and cell death program activation (e.g., the apoptotic cascade) [4]. Anoikis, a greek word meaning loss of “home” or “homelessness”, is a specific form of caspase-mediated programmed cell death that occurs when the cell cycle is arrested due to the loss of the normal cell–matrix interactions. Anoikis represents an essential defense for the organism by inhibiting detached cells’ re-adhesion to new matrices in inappropriate environments and their dysplastic growth. As a result, the resistance to anoikis program execution, an emerging hallmark of cancer cells, is a crucial step in the acquisition of malignancy as cancer cells can migrate throughout the body, spread into the surrounding tissues and distant organs, and grow metastatically [5]. A main role in anoikis is played by Integrins, surface receptors composed of two non-covalently associated α and β subunits that sense the extracellular matrix (ECM) and connect it to the cytoskeleton, therefore regulating many important cell processes. In adherent cells, in fact, cell-specific activation of Integrins and their downstream signaling mediators are responsible for anoikis protection by transducing signals, which are necessary for cell proliferation, survival and migration, from the extracellular matrix (ECM) to the cell [5,6]. Moreover, unligated Integrins can also act as cell-death promoters, through a process named ‘integrin-mediated death’ (IMD). In neuroblastoma cells, anoikis obtained by unligated integrin-induced caspase 8 activation prevents metastasis and the caspase-8 suppression is associated with increased invasive capacity in vivo [7].

In addition, a body of literature data highlights that epigenetic aberrations such as alterations in chromatin state, DNA methylation, histone modifications and the posttranscriptional regulation of gene expression by miRNAs play a crucial role in the tumor suppressor silencing, oncogene activation, or in the cell fate transitions, thus giving rise to all of the classic hallmarks of cancer [8,9,10].

All these aspects of cancer biology are widely regulated by environmental and lifestyle factors including physical activity, nutrition, dietary supplements (herbs, nutraceuticals and phytochemicals) and synthetic agents. Many natural bioactive compounds have demonstrated their capability in counteracting tumorigenesis as well as tumor progression in various cancer types, through their impact on genomic instability, tumor-promoting inflammation, dysregulated metabolism, immune system evasion and epigenetics [4,11,12]. These findings have encouraged the research and the use of diet and/or nutraceutical supplementation as new integrated therapies in primary cancer prevention, in improving the efficiency of chemotherapy and in survivors’ management [13].

Cruciferous vegetables (broccoli, broccoli sprouts, cabbage or kale), a rich source of glucosinolates, which are metabolized to isothiocyanate compounds (ITCs), exert many anticancer effects both in vitro and in vivo models [14]. Sulforaphane (1-siothiocyanato-4-methylsulfinylbutane, SFN), obtained from myrosinase-catalyzed hydrolysis of glucoraphanin (GR), is one of the major ITCs that regulates carcinogenesis through the targeting of multiple mechanisms within the cell [15].

Notably, SFN exerts its effects through the Nrf2-mediated induction of phase II detoxification enzymes, with the suppression of oxidative stress-induced DNA damage, inhibition of proliferation, histones modulation, anti-inflammatory and pro-apoptotic activity [15,16,17,18,19]. Moreover, SFN is able to enhance the anticancer activity of chemotherapeutic agents [20,21] and to renew anoikis resistance in many common cancers, e.g., prostate, lung and colorectal cancers [22,23,24].

Although SFN is water-soluble and well absorbed after oral administration both in humans and animals [25,26], the SFN bioavailability from cruciferous vegetables varies widely based upon many factors, mainly stemming from the conversion rate of GR to SNF by the enzyme myrosinase. This plant enzyme is commonly segregated from the glucosinolates until the cells are disrupted and is also produced by gut microbiome [27]. The inactivation of myrosinase, as it may be in extract supplementation, significantly reduces SFN absorption [28].

Sita et al. (2018) reviewed a number of studies reporting the use of adjunctive SFN therapy in GBM for better targeting resistance and synergistically complementing and improving standard treatments as it induces apoptosis, and inhibits both growth and invasion of GBM cells [3].

Moreover, we chose the 1321N1 cell line with mutated *cellular tumor antigen p53* (mut p53 R213Q) as a model, because the *p53 gene* is mutated in approximately 40% of astrocytic tumours and dissection of the molecular pathogenesis of astrocytic tumors has identified defects in the p53 pathway as one of the most common molecular alterations observed in human astrocytoma involved in both the early transforming events and progression from low-grade to high-grade neoplasms. The functional elimination of these critical cell cycle regulatory and apoptosis-inducing factors is believed to contribute to the aggressive and invasive nature of these tumors [29].

For the first time, we utilized Rapha Myr^®^, a novel blend of broccoli seed extract (*Brassica Oleracea* s.e., Sulforaphane glucosinolate titer 11%) plus active myrosinase, to treat the human astrocytoma cell line (1321N1). In the present study, we investigated the anticancer activity of Rapha Myr^®^, demonstrating that Rapha Myr^®^ elicited antiproliferative effects by inducing cell cycle arrest, oxidative stress and genotoxicity accompanied by global DNA hypermethylation and increased levels of DNA methyltransferase 1 (DNMT1), and changes in sirtuins’ expression and activity. Moreover, after Rapha Myr^®^ treatment, the cells lose migratory and proliferative properties as proved by cell migration inhibition, cytoskeleton network destructuration, and the blocking of integrin α5 translocation and expression. As result, the cell cycle is arrested and an anoikis-like death is induced via p53-independent mechanisms and under the epigenetic control of gene expression.

## 2. Results

### 2.1. Antioxidant Capability of Rapha Myr^®^

**Figure 1:**
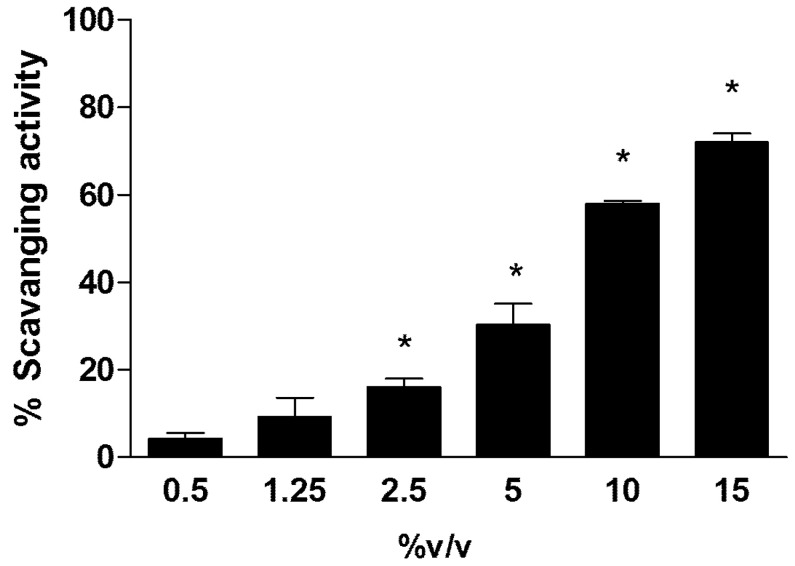
Antioxidant capability of Rapha Myr^®^ extract (0.5–1.25–2.5–5–10–15% *v/v*) evaluated by DPPH assay. The scavenging activity was expressed as percentage decrease in absorbance with respect to control. Values are mean ± SD of three experiments in triplicate. * *p* < 0.05 vs. control.

The results of antioxidant capability refer to different concentrations of Rapha Myr^®^ extract measured by DPPH assay and are reported in Figure 1. The data shows that an important antioxidant activity is exhibited only at a concentration of Rapha Myr^®^ higher than 2.5% *v/v*.

### 2.2. MTT Assay, Cell Morphological Analysis, DNA Integrity and Redox Status

We compared the cytotoxicity of Rapha Myr^®^ extract in tumour and non-tumour cells by evaluating the IC50 values and cell morphology in 1321N1 (human astrocytoma cell line), U87 (human glioblastoma cell line), SHSY5Y (human neuroblastoma cell line) and HFF1 (Human Foreskin Fibroblast cell line).

**Figure 2:**
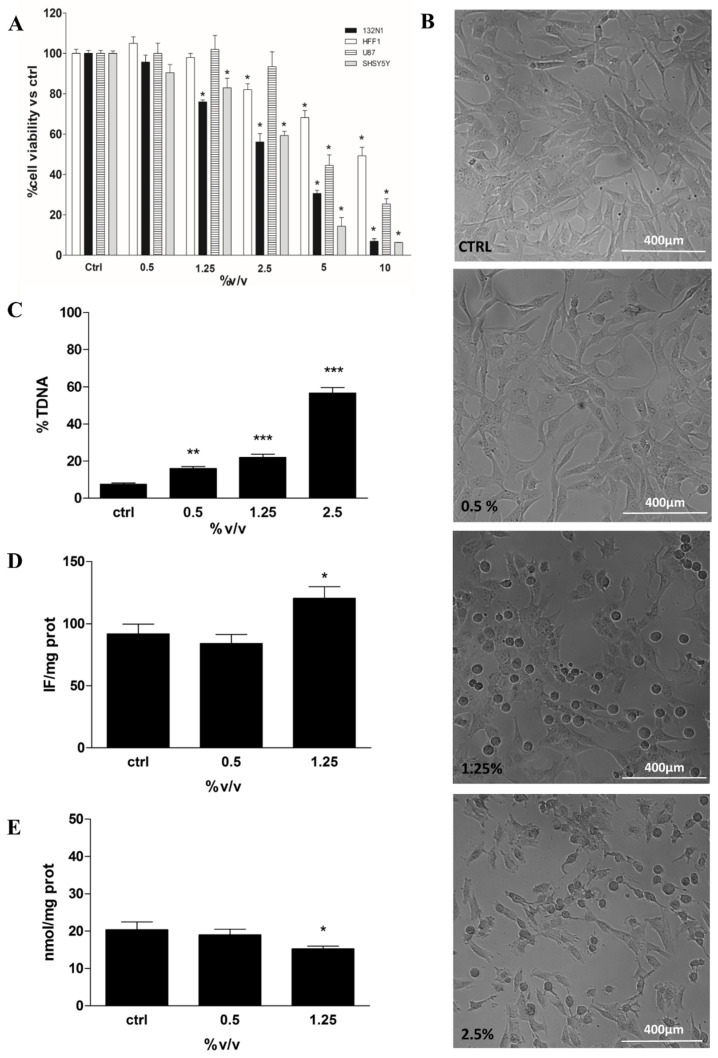
Cell viability, cell morphological analysis, DNA integrity and Redox Status in 1321N1 cells untreated and treated for 24 h with different concentrations of Rapha Myr^®^ extract depending on each cell-based assay protocol. (**A**) Cell viability in HFF1,1321N1, SHSY5Y and U87 cells evaluated by MTT assay. The results were expressed as a percentage of cell viability compared to untreated control viable cells, which had a value equal to 100%. (**B**) Representative images of 1321N1 cell morphology acquired by optical inverted light microscopy. Original magnification 10×, scale bar 400 µm. (**C**) DNA damage was evaluated by alkaline comet assay. The results were expressed as the percentage of DNA present in the comet tail (%TDNA). (**D**) Intracellular reactive oxygen species (ROS) levels evaluated by using 2′,7′-dichlorofluorescein diacetate (DCFH-DA); the results were expressed as intensity of fluorescence (IF) per mg protein. (**E**) Non-protein thiol levels in 1321N1 cells; the results were expressed as nmol GSH/mg proteins. All values are mean ± SD of three experiments in triplicate. * *p* < 0.05 vs. control; ** *p* < 0.01 vs. control; *** *p* < 0.001 vs. control.

MTT assay was performed on all cell lines treated with Rapha Myr^®^ extract (0.5–10% *v/v*) for 24 h. The treatment of both glioma cells (1321N1 and U87) and neuroblastoma cells (SHSY5Y) with Rapha Myr^®^ extract results in various degrees of inhibition of cell viability based on the extract concentration. The results highlight that Rapha Myr^®^ extract mainly reduces, in a dose-dependent manner, the viability of both 1321N1 astrocytoma cells and SHSY5Y neuroblastoma cells compared with U87 glioblastoma cells (Figure 2A). The IC50 values of Rapha Myr^®^ extract are 2.95%, 2.72% and 4.66% in these tumour cell lines after 24 h, respectively. The IC50 value of Rapha Myr^®^ extract is 9.93% in the HFF1 non-tumour cell line, suggesting that Rapha Myr^®^ is cytotoxic towards cancer cells.

Moreover, a morphological change in 1321N1 was induced by exposure to Rapha Myr^®^ extract (0.5–1.25–2.5% *v/v*) for 24 h (Figure 2B). Mainly at 1.25 and 2.5% *v/v*, the cells did not grow on the plate surface uniformly but were dispersed, showing an altered shape and a reduced proliferation compared with the control cells. Rapha Myr^®^ 2.5% induced the death of cancer cells, with many cells floating in the medium. Besides, the inverted light microscope images of SHSY5Y and U87 tumour cells show dose-dependent morphological modifications (rounding, shrinkage and reduced adhesion) upon 24 h exposure to Rapha Myr^®^ (0.5–1.25–2.5% *v/v*), which are more evident in SHY5Y (Figures S1 and S2). Conversely, the viability, cell morphology and adhesion of HFF1 normal cells were not affected by the Rapha Myr^®^ concentrations that were effective on cancer cells (1321N1 ≥ SHSY5Y >> U87 > HFF1) (Figure S3).

Taking in to account these preliminary results and considering drug resistance of brain tumours harbouring mutated p53 [29], we selected the human astrocytoma 1321N1 cell line to test the biological effects of Rapha Myr^®^.

The genotoxicity of Rapha Myr^®^ extract (0.5–1.25–2.5% *v/v*) was evaluated on 1321N1 by alkaline comet assay in order to assess whether our extract could cause DNA damage responsible for its anti-tumour effects. After 24 h of treatment, a dose-dependent increase in DNA damage was observed, being 16.05%, 22.15% and 56.66%, respectively (Figure 2C).

The involvement of redox status has been investigated by the determination of both ROS (DCFDA assay) and non-protein thiols levels (DTNB method). The data indicates that Rapha Myr^®^ extract increases ROS production and concurrently decreases GSH levels in human astrocytoma 1321N1 cells (Figure 2D,E).

### 2.3. Cell Migration Inhibition and Cytoskeleton Structure Alteration

**Figure 3:**
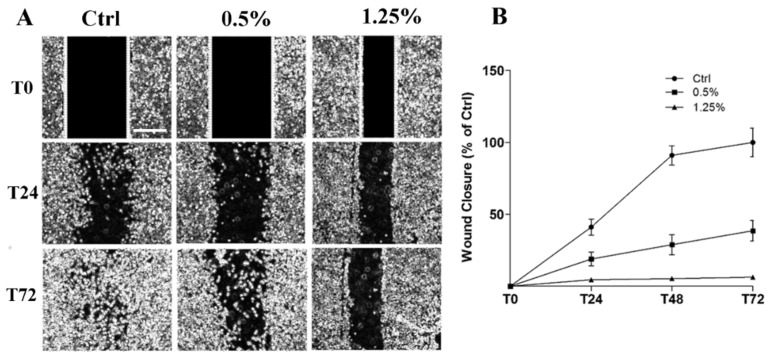
Cell migration evaluated by Wound Healing assay in 1321N1 cells untreated and treated with different concentrations of Rapha Myr^®^ extract (0.5 and 1.25% *v/v*). (**A**) Phase contrast microscope images at 0, 24 and 72 h post-scratch. Original magnification 4×, scale bar 1000 µm. (**B**) The migratory capacity of 1321N1 cells was measured at 24, 48 and 72 h as the percentage of wound closure compared to time 0 h when the scratch was made.

Figure 3A shows the results of cell migration analysis by wound healing assay on 1321N1 untreated and treated with different concentrations of Rapha Myr^®^ extract for 72 h. The width and the closure capacity of the wound, relative to initial width, was measured at different time points (0, 24, 48 and 72 h). The results point out that wound healing capacity was severely reduced in Rapha Myr^®^-treated cells compared with untreated control cells. Particularly, Rapha Myr^®^ extract 1.25% *v/v* totally inhibited the wound closure. (Figure 3B).

**Figure 4:**
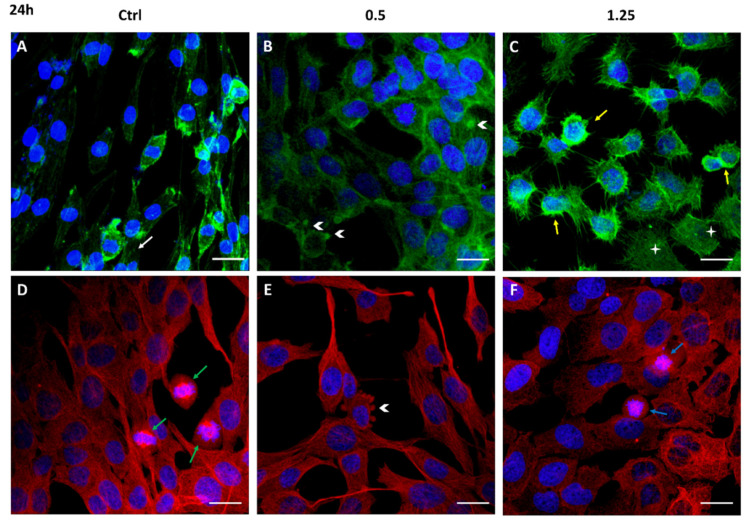
Cytoskeleton structure analysis of 1321N1 cells untreated (**A**,**D**) and treated with 0.5% (**B**,**F**) and 1.25% *v/v* (**C**,**D**) Rapha Myr^®^ extract for 24 h. Representative IF images for actin stained with FITC-Phalloidin (green; **A**,**B**,**C**), microtubules stained with anti–α-tubulin antibody (red; **D**,**E**,**F**) and nuclei stained with DAPI (blue), a merge was made. White arrows: stress fibers; yellow arrows: cellular cortex; arrowheads: blebs; green arrows: mitosis; blue arrows: abnormal mitosis. Scale Bar: 20 μm.

**Figure 5:**
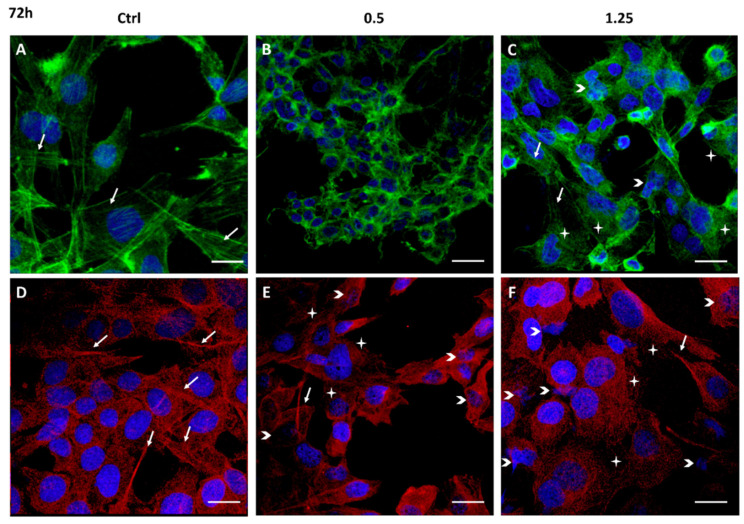
Cytoskeleton structure analysis of 1321N1 cells untreated (**A**,**D**) and treated with 0.5% (**B**,**E**) and 1.25% *v/v* (**C**,**F**) Rapha Myr^®^ extract for 72 h. Representative IF images for actin stained with FITC-Phalloidin (green; **A**,**B**,**C**), microtubules stained with anti–α-tubulin antibody (red; **D**,**E**,**F**) and nuclei with DAPI (blue); a merge was made. White arrows: stress fibers in green fluorescent images and bundles of microtubules in red fluorescent images; arrowheads: small or misshapen nuclei; stars: disorganized actin or microtubule network. Scale Bar: 20 μm.

Cell migration is a complex multi-step process, also driven by cytoskeleton proteins, utilized by cancer cells to invade and metastasize other tissues. The structural analysis of the cytoskeleton by immunofluorescence after 24 h and 72 h exposure to Rapha Myr^®^ (0.5% and 1.25%) is reported in Figure 4 and Figure 5. The cytoskeleton of untreated astrocytoma cells shows microfilaments organized in stress fibers, filipodia and lamellipodia, and in the cortex (Figure 4A and Figure 5A). Microtubules are assembled into a fine fibrillary network, and some normal mitotic spindles are also visible (Figure 4D). At 72 h, the cell monolayer still shows an apparently continuous microtubular network that is an evident sign of cell–cell adhesion (Figure 5D).

After 24 h of treatment with Rapha Myr^®^ 0.5% *v/v* and 1.25% *v/v*, the cell architecture is strongly altered. In particular, at 0.5% *v/v* the cell cortex appears more evident around the small nucleus while microfilaments are depolymerized in the cytoplasm of cells flattened onto substratum (Figure 4B). The microtubular network is compact, forming thick bundles above all in the cytoplasmatic protrusion (Figure 4E). The plasma membrane shows some blebs fluorescent in green (FITC-Phalloidin) or red (Alexa Fluor 594).

After treatment with Rapha Myr^®^ 1.25% *v/v*, the microfilaments thicken in the cell cortex and in the numerous thin filopodia protruding radially from the cell body, indicating that the cell migratory capacity and the adhesion onto the substrate are impaired. Spread cells show a pale-green cytoplasmic fluorescence, an evident sign of F-actin depolymerization (Figure 4C). Conversely, microtubules are irregularly localized in the cytoplasm and around the nucleus (Figure 4F). The inhibition of microtubule function results in abnormal mitotic spindles (Figure S4), multinucleated cells and decondensed nuclei (Figure 4F).

All concentrations of Rapha Myr^®^ extract cause strong changes in the cytoskeleton at 72 h (Figure 5B,C,E,F). At 0.5%, microfilaments and microtubules appear strictly condensed around nuclei that are smaller than control cells (Figure 5B,E). After Rapha Myr^®^ 1.25%, the actin cortex is well-organized only in rounded cells while the green fluorescence is weak and widespread in the cytoplasm of spreading cells (Figure 5C). The microtubule network is totally disarranged and tubulin appears like bright fluorescent spots instead of microtubules (Figure 5F). Moreover, nuclei of different shapes and sizes were observed and nuclear chromatin fragmentation was detected, a clear feature of apoptosis (Figure S5).

### 2.4. ECM–Cell Adhesion and Integrin α5 Expression as Hallmark of Anoikis

**Figure 6:**
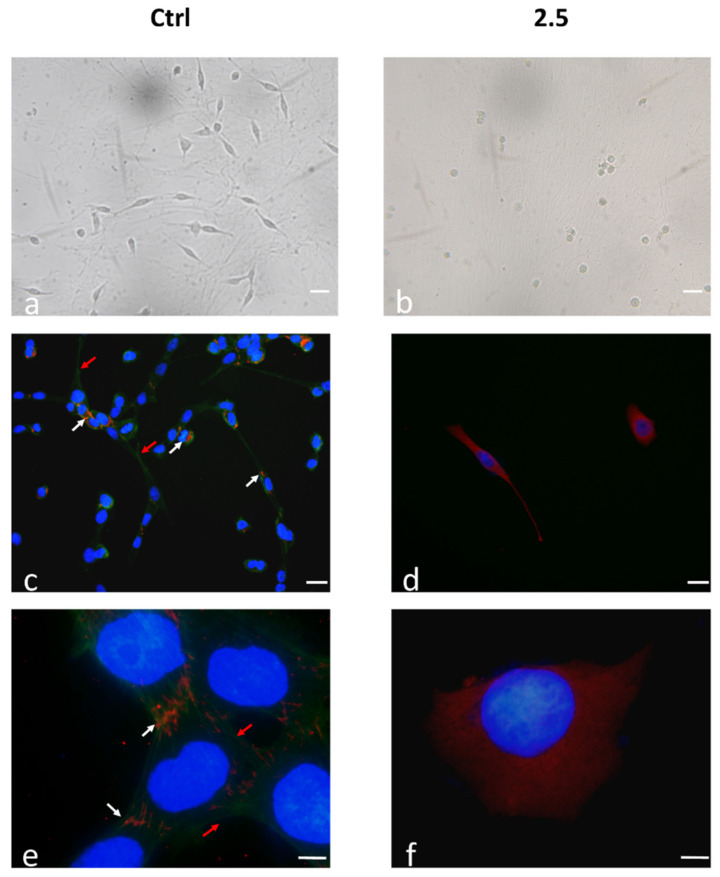
Cell morphology of 1321N growth on the extracellular matrix (ECM) coating untreated (**a**) and treated (**b**) with 2.5% *v/v* of Rapha Myr^®^ for 24 h. Images were acquired by optical inverted light microscopy. Original magnification 25×, scale bar 10 µm. Distribution of Integrin α5 (red fluorescence) and microfilaments (green fluorescence) in 1321N1 cells on ECM coatings. Control cells: (**c**,**e**); 2.5% Rapha Myr^®^ extract: (**d**,**f**). Nuclei was stained with DAPI and a merge was made. White arrows: α5 Integrins; red arrows: stress fibers. Original magnification: 20×, scale bare 20 μm (**c**,**d**); 100×, scale bar 1 μm (**e**,**f**).

Integrins and their interaction with ECM fibrous components play a pivotal role in regulating the death/survival balance and anoikis, an apoptotic process also activated by unligated-integrins [6]. 1321N1 cell adhesion on DPSC-derived extracellular matrix (ECM) after treatment with 2.5% *v/v* Rapha Mir^®^ was evaluated. Figure 6 shows two representative images of 1321N1 cells (control and 2.5%) after 24 h of culture on the ECM. Control cells show a more fibroblastic-like shape, due to the effect of the matrix components that promote directional orientation along the matrix fibrils (Figure 6a). Conversely, few cells are attached and most of them are roundish and in suspension in the 2.5% Rapha Mir^®^-treated sample (Figure 6).

To better investigate cells’ adhesion of 1321N1 onto the ECM, the behavior of both Integrin α5 and microfilaments after 72 h of treatment with 2.5% Rapha Mir^®^ were evaluated by double indirect immunofluorescence (Figure 6). In untreated 1321N1 (Figure 6c,e), Integrin α5 is clearly evident at both magnifications, while microfilaments are hardly visible at 20×. The receptor for fibronectin shows the characteristic rod-like structure, all oriented in the same direction corresponding to the microfilaments’ arrangement that is now observable as evident stress fibers; they are also localized on the cell surface and adhere on the extracellular matrix coating, indicating a correct process of adhesion to the ECM fibronectin (Figure 6e). As previously mentioned, Rapha Mir^®^ depolymerizes microfilaments, in fact, we observe an almost total absence of green fluorescence and an intense red fluorescence around the nucleus as well as in the cytoplasmatic body in Rapha Mir^®^-treated cells (Figure 6d,f). The absence of stress fibers does not allow integrins translocation toward the peripheral region of the cell, resulting in a poor and weak adhesion. Integrins, not being unable to move, remain blocked in the cytoplasm.

**Figure 7:**
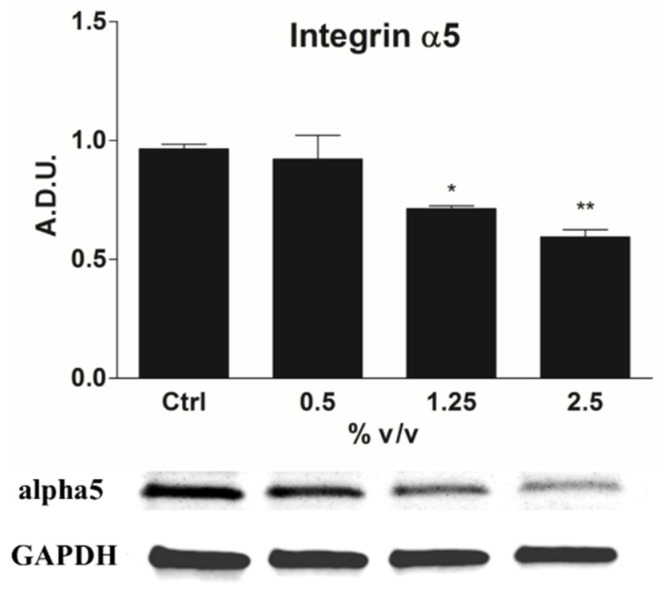
Western blot of integrin α5. Bar graph shows the relative expression of the respective protein level. Representative immunoblotting image of integrin α5 expression. All values are mean ± SD of three experiments in triplicate. * p < 0.05; ** *p* < 0.01 vs. control.

Significant changes in cell migration, cytoskeleton structure and adhesion to ECM are observed in 1321N1 cells treated with Rapha Myr^®^. Therefore, the expression of integrin α5, which is associated with adhesion to fibronectin, was examined by immunoblotting. Our data points out that Rapha Myr^®^ induces a dose-dependent decrease in α5 integrins mainly after the 2.5% treatment, which shows the lowest alpha5 expression (Figure 7).

### 2.5. Cell Cycle Progression, Apoptosis and Related Protein Expression

**Figure 8:**
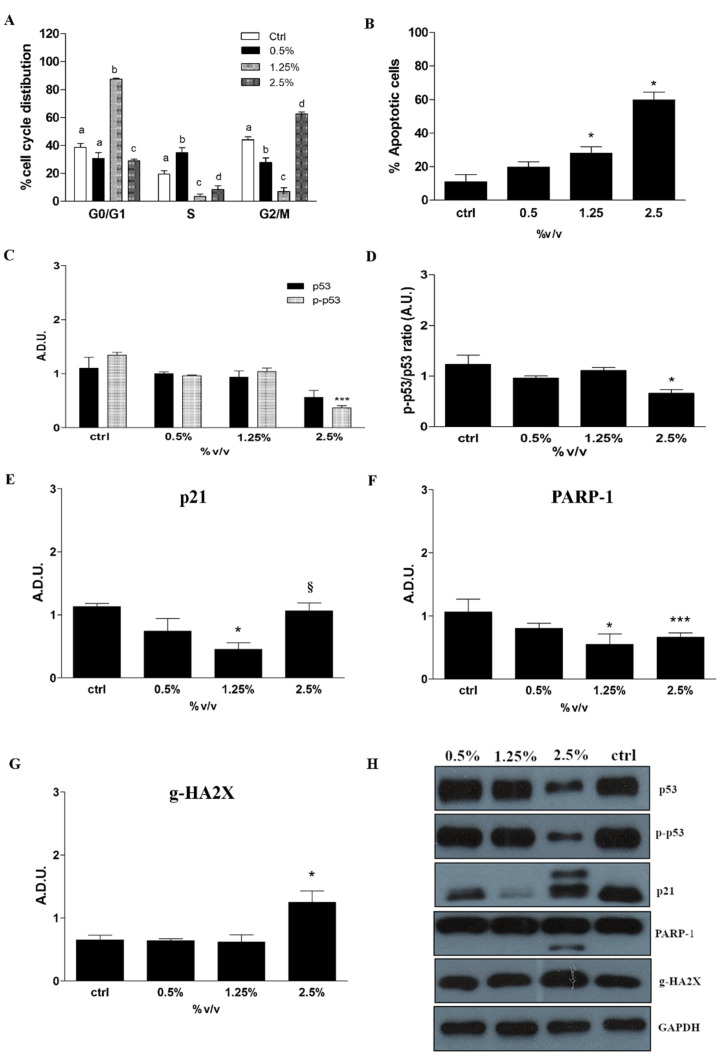
Cell cycle analysis, apoptosis determination and protein expression levels in 1321N1 cells untreated and treated with different concentrations of Rapha Myr^®^ extract (0.5–1.25–2.5% *v/v*) for 24 h. (**A**) Cell cycle analysis performed by flow cytometry using propidium iodide DNA staining. The graph shows the percentage of 1321N1 cell distribution in different cell cycle phases. Bars that do not share the same letter are significantly different. (**B**) Apoptosis measured by flow cytometry and Annexin-V/PI co-staining. The bar graph shows the percentage of apoptotic cells (Annexin-V-positive, PI-negative and -positive). Western blot of p53 and p-p53 (**C**), p-p53/p53 ratio (**D**), p21 (**E**), PARP1 (**F**) and γH2AX (**G**). Each bar graph shows the relative expression of the respective protein level. (**H**) Representative immunoblotting image of each protein examined. All values are mean ± SD of three experiments in triplicate. * *p* < 0.05 vs. control; *** *p* < 0.001 vs. control; ^§^ *p* < 0.01 vs. 1.25%.

The cell cycle analysis by flow cytometry shows a different cell distribution after Rapha Myr^®^ treatment for 24 h (Figure 8A and Figure S6). A significant blockage in the S phase at 0.5% *v/v* and in G0/G1 phase at 1.25% *v/v* is observed. However, both treatments induce a progressive reduction in cells in the G2/M phase compared to the untreated cells, with a decrease of 16.5 and 36.93% in the DNA content, respectively. Conversely, the treatment with Rapha Myr^®^ extract 2.5% *v/v* induces an arrest in the G2/M phase with an increase of 18.53% compared with control cells (Figure 8A and Figure S6).

The induction of apoptosis evaluated cytofluorimetrically by Annexin V/PI staining shows that the percentage of apoptotic cells (early + late) is significantly upregulated after 24 h in the Rapha Myr^®^-treated 1321N1 cells (Figure 8B and Figure S7). The percentages of early apoptotic cells after the treatment with Rapha Myr^®^ extract 0.5, 1.25 and 2.50% *v/v* are 17.9, 22.7, and 35.1%, respectively, as compared to the control (8.2%).

Western blot analysis shows that the expression of some proteins involved in the cell cycle regulation is modulated by Rapha Myr^®^ treatment for 24 h. We observe a decrease in the mutated p53 R213Q, phospho-p53 (Ser15) and their ratio (pp53/p53), which is more significant at 2.5% *v/v* (Figure 8C,D). The treatment also results in a reduction in cyclin-dependent kinase inhibitor 1 level (p21) that is significant only at 1.25% *v/v*. Instead, the treatment with Rapha Myr^®^ extract 2.5% *v/v* increases the p21 protein levels more significantly compared with 1.25% (Figure 8E).

The analysis of the protein expression of Poly [ADP-ribose] polymerase 1 (PARP-1) and phosphorylated Histone H2AX (γH2AX), two proteins involved in DNA damage repair and apoptosis, shows that the treatment with Rapha Myr^®^ extract 2.5% *v/v* induces the cleavage of the apoptotic marker PARP-1, other than a significant upregulation of γH2AX (Figure 8F,G).

### 2.6. Epigenetic Modulation

**Figure 9:**
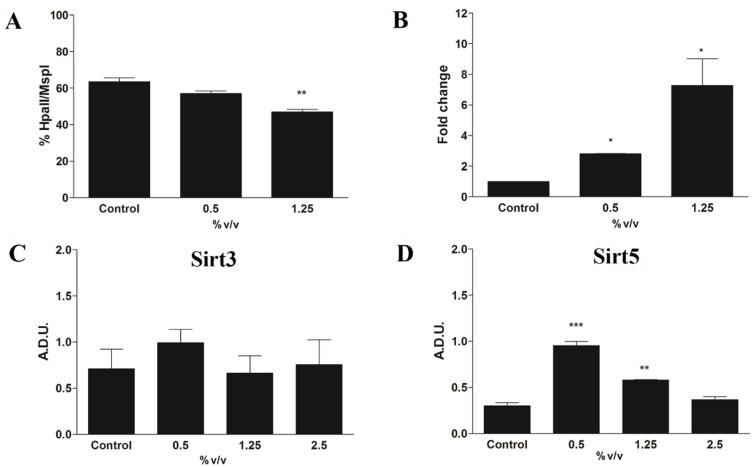

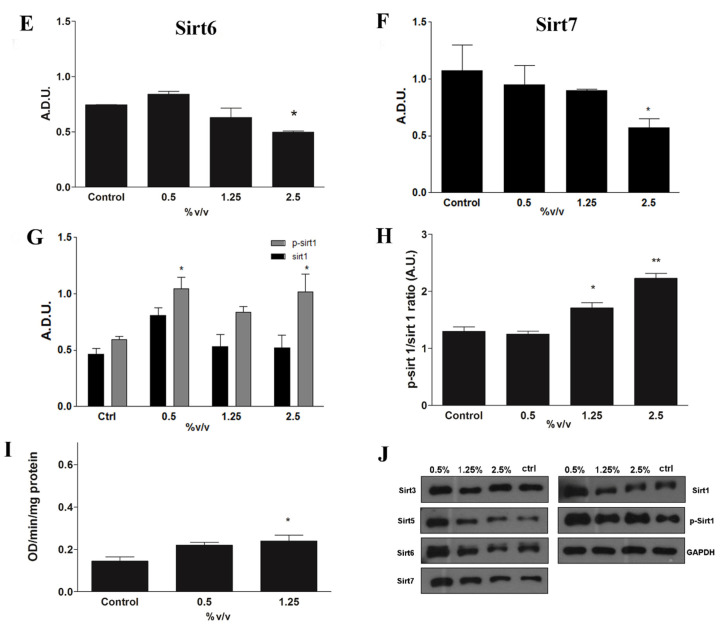
Epigenetic modulation in 1321N1 cells untreated and treated for 24 h with different concentrations of Rapha Myr^®^ extract depending on each cell-based assay protocol. (**A**) Global DNA methylation evaluated by Methy-sens comet assay. (**B**) Changes in mRNA level of DNA methyltransferase1 (DNMT1). Western blot of mitochondrial Sirt3 (**C**) and Sirt5 (**D**). Western blot of nuclear Sirt6 (**E**), Sirt7 (**F**), Sirt1 and pSirt1 (**G**), and pSirt1/Sirt1 ratio (**H**). Each bar graph shows the relative expression of the respective protein level. (**I**) Nuclear sirtuins activity expressed as OD/min/mg proteins. (**J**) Representative immunoblotting image of each protein examined. All values are mean ± SD of three experiments in triplicate. * *p* < 0.05 vs. control; ** *p* < 0.01 vs. control; *** *p* < 0.001 vs. control.

Methy-sens comet assay shows that Rapha Myr^®^ extract is a DNA hypermethylating agent in 1321N1 cells as judged by decreased % TDNA levels after incubation with HpaII, an enzyme unable to cut DNA when the second cytosine is methylated (Figure 9A). Changes in global DNA methylation are consistent with corresponding changes in the mRNA level of DNMT1, which is upregulated after treatment with Rapha Myr^®^ extract (Figure 9B).

In Figure 9C–I are reported the results obtained by Western blot for the nuclear and mitochondrial sirtuins. The data shows that Rapha Myr^®^ extract, after 24 h, is able to influence most of these deacetylases, except the mitochondrial Sirt3 (Figure 9C), which is unmodified by all doses utilized. An increase in mitochondrial Sirt5 levels (Figure 9D) is observed in both 0.5 and 1.25% treated cells, whereas 2.5% treatment reduces its expression, taking it back to the control value. In addition, Rapha Myr^®^ extract 2.5% *v/v* reduces both the Sirt6 and Sirt7 expression levels (Figure 9E,F). Although all of the treatments do not significantly modify the Sirt1 expression level, the content of phosphorylated Sirt1 is upregulated by Rapha Myr^®^ extract and also the pSirt1/Sirt1 ratio is significantly increased (Figure 9G,H). These changes in the expression of nuclear sirtuins are also accompanied by a significant increase in their enzymatic activity at 1.5% (Figure 9I).

## 3. Discussion

Cancer treatment is still a major challenge; in fact, although chemotherapy/radiotherapy are the major therapeutic approaches, their efficiency is limited by toxicity and poor utilization of personalized oncology therapies [30,31]. Moreover, the “resistance to anoikis”, a peculiar kind of apoptosis characterized by anchorage-independent growth of cancer cells, is now considered a hallmark of cancer cells [32]. Surprisingly, literature data suggests that both the introduction of appropriate nutrients, both by diet or as supplements, and improving lifestyle could prevent 30–40% of cancers, improve responses to conventional therapies and reduce their adverse effects [33]. Among the different valuable nutrients, sulforaphane (SFN) has largely demonstrated its beneficial potentiality in prevention and in multistage cancer development, by affecting important cellular mechanisms including the epigenetic pathway modulation, the sensitivity restoration to anoikis in some cancer cell types and animal models, the alteration in cell cycle control with p21 up-regulation and a block in the PI3K/Akt signaling pathway [12,15,21,22,34]. Several studies also highlight the potent activity of SFN versus the most malignant brain tumor, glioblastoma, targeting apoptosis and cell survival pathways [3].

At the beginning, we carried out the evaluation of viability and cell morphology on three brain tumor cell lines (1321N1, U87 and SHSY5Y) and one normal cell line (HFF1), demonstrating that Rapha Myr^®^ is safer for normal cells and more active against tumor cells, mainly to both astrocytoma (1321N1) and neuroblastoma (SHSY5Y) cells. Although a lot of studies report the use of adjunctive SFN therapy for targeting glioblastoma, no evidence supports its use in astrocytoma treatment [3]. Moreover, we decided to choose the 1321N1 cell line as a model with mut p53 R213Q since about 40% of astrocytic tumours express mutant forms of p53 but its role in the molecular pathogenesis of these tumours deserves further study [29].

To the best of our knowledge, this is the first study that investigates 1321N1 human astrocytoma cell lines harbouring mut p53, the effect of Rapha Myr^®^, a novel blend of sulforaphane and hydrolytic enzymes myrosinase capable of improving isothiocyanates’ bioavailability [15]. Our experimental setup tackled some aspects implicated in the potential chemotherapeutic activity of Rapha Myr^®^, focusing on some pathways involved in cytotoxicity and DNA damage, as well as cell cycle arrest, disarrangement of cytoskeleton and epigenetic modulation.

In this study, we found that Rapha Myr^®^ may be a promising anticancer compound for astrocytoma mostly due to anoikis induction. Rapha Myr^®^ exerted it effects in a dose-dependent manner, particularly, after 2.5% treatment most cells changed their morphology, floated in the medium and then underwent apoptosis. Rapha Myr^®^-induced anoikis may be associated with cytoarchitecture alteration at 1.25%, as shown by immunofluorescence analysis. In astrocytoma cells, we demonstrated that Rapha Myr^®^ provoked protein–cytoskeletal reorganization with growth arrest, shape modifications and inhibition of migration capability, probably resulting from its effects on the microtubule polymerization by interaction with cysteine residues of tubulin [35] and on the Rho pathway alteration leading to the disarrangement of the actin-cytoskeleton [36]. Our results agree with previous data reporting that sulforaphane causes changes in the shape and migration processes of cells, altering the enormous dynamism of actin, and influences microtubule polymerization and depolymerization, regulating cell polarity, mitosis and cell motility. In addition, sulforaphane acts as an inhibitor of microtubule polymerization in rapidly growing colon cancer cells with a block of proliferation and cell cycle arrest [37].

It is well known that tumour cells escape anoikis through diverse mechanisms such as changes in the integrins’ repertoire, the activation of oncogenes and pro-survival signal pathways, or upregulation of growth factor receptor and their transduction pathways. Notably, integrins play a role in cell growth, differentiation, and death by regulating the interaction between cell and ECM [5,6]. In GBM α6β4, α5β1, αvβ6, and αvβ3 are upregulated and correlated with poor patient survival [38]. Besides, α5β1 integrin reduces GBM cell proliferation when inhibited [39] and was shown to mediate EMT in GBM cells [40] which is one of the strategies adopted by cancer cells to avoid anoikis.

In the extracellular matrix, the molecules of fibronectin assemble into fibrils only on the cell surface in a process guided by additional proteins, especially Integrin α5β1, which mediates the interactions between the extracellular fibronectin fibrils and intracellular actin filaments and promotes fibronectin fibril polymerization and matrix assembly [41].

In our study, the distension of untreated 1321N1 cells on ECM-coated surface was clearly mediated by cytoskeletal components, mainly microfilaments. At the same time, integrin α5 allowed cells to anchor onto ECM by arranging correctly on the entire cell surface. Unlike the untreated cells, the exposure to Rapha Myr^®^ 2.5% for 72 h inhibited integrin α5 translocation from the cell center towards the cell surface, and reduced its expression in a dose-dependent manner. We can speculate that the destructive effect of Rapha Myr^®^ on actin polymerization may be responsible for the alteration in adhesion molecules’ pattern, which is associated with the reduced adhesion to ECM due to the absence of microfilaments organized in stress fibers, as demonstrated by immunofluorescence. Consequently, the loss of adhesion may lead to cell cycle arrest and anoikis of 1321N1, as observed by us.

We also found a reduction in proliferation and cell cycle block as a consequence of genomic DNA damage. Consistent with this, we observed a high DNA fragmentation associated with a moderate cleavage of PARP-1, a hallmark of apoptosis, and the increase in γH2AX level indicating that Rapha Myr^®^ increased double strand break damage. Many anticancer therapies (i.e., radiation or drugs) induce DNA damage directly or interfere with DNA metabolism achieving DNA double-strand breaks (DSBs) responsible for apoptotic cell death. γH2AX protein is one of the main actors in DNA repair and damage response (DDR) pathways forming a focus at the DSB site. PARPs are also activated by DNA damage including DNA single- and double-strand breaks [42].

One of the mechanisms associated with SFN cytotoxic activity in HeLa, HCT116 and HT29 cell lines is its genotoxicity [43,44]. For instance, DNA damage, ATM and Chk2 kinase activation, γH2AX nuclear foci formation and subsequent G2/M cell cycle arrest have been observed in SFN-treated prostate cancer cells [45,46]. Similarly to these previous studies, here we also documented a cell cycle arrest at the G2/M phase following treatment with 2.5% Rapha Myr^®^. All these alterations triggered proapoptotic signals in a concentration-dependent way, which results in apoptotic cell death, as indicated by the increase in percentage of apoptotic 1321N1 cells exposed to 2.5% of Rapha Myr^®^. Furthermore, the investigation of the molecular mechanism involved in 1321N1 cell cycle and cell death by studying expression levels of p53, the “guardian of genome”, and its downstream effector p21, the cyclin-dependent kinase inhibitor, allows us to demonstrate that Rapha Myr^®^ would seem to regulate both these cellular processes in a p53-independent manner. *P53 gene* is frequently mutated in human cancer and the mutant p53 proteins not only lose their tumor-suppressor function, but may also gain new oncogenic functions and promote tumorigenesis [47]. This potential mechanism activated by Rapha Myr^®^ can be related to the mutation state of p53, in fact, it is reported that the mutation c.638G > A (p.R213Q) is present in 1321N1 cells, severely compromising the capability to induce p53-regulated genes; mainly harbouring amino acid changes in its DNA-binding domain, it does not transactivate p21 [48,49]. In our model, the oncogenic function of mutant p53-R213Q may promote both cell survival and anoikis resistance of 1321N1 cancer cells, also being less efficient in binding the consensus sequence in the p21 gene-regulatory region [49]. This supports our result showing a reduction in p21 at 1.25% concentration of Rapha Myr ^®^, despite the presence of p53 (Figure 8H). Rapha Myr^®^.2.5% also decreases the concentration of the oncogenic p53 mutant protein and several papers report that SFN-mediated apoptosis was independent of a mutated p53 status [50]. At the same time, Rapha Myr^®^.2.5% increases the p21 levels with respect to 1.25%, thus inhibiting survival by induction of cell cycle arrest, apoptosis and anoikis. Our data partially agrees with previous studies demonstrating that p53 and p21 are involved in cell detachment and anoikis resistance, as well as that the anoikis escaping from cancer cells is favored either by suppression of p53 activation or p53 mutations [49,50,51]. In addition, Tsai and colleagues showed that A549 cells with wild-type p53 are more sensitive to SFN-induced anoikis than p53-mutant CL1–5 cells and also demonstrate that p53 and its downstream effector p21 are negative regulators of anchorage-independent growth [22]. However, p21 can be transactivated by other transcription factors than p53. In our model, it seems likely that other factors might contribute to p21 regulation via different mechanisms, independent of p53, to convey different oncosuppressive signals able to hinder aggressiveness in human cancers [52]. We think that the effects observed may not only be linked to a reduction in p53 mutant form or dephosphorylation of p53 influencing other p53 targets, but also to direct and/or indirect action of Rapha Myr^®^; for instance, its impact on cellular oxidative balance with the increase in ROS production and the reduction in free thiols groups, as observed by us. Many authors showed that p53 mutant isoforms maintain high ROS levels in cancer cells through a coordinated control of various redox-related enzymes and signaling pathways, thus favoring cancer cell proliferation [53,54]. We can speculate that 131N1 cancer cells expressing mutant p53 proteins can be significantly more sensitive to a pro-oxidant environment, thus Rapha Myr^®^ induced an anoikis-like death by provoking redox unbalance following modulation of both mutant p53 isoform and p21 expression. Intriguingly, this cellular context driven by inhibition of mutant p53 might be responsible for the cytoskeleton disarrangement and inhibition of cell migration contributing to the promotion of anoikis-like apoptosis and reducing metastatic spread.

A significant amount of evidence indicates that SFN also promotes secondary chemoprevention by targeting the transcription of apoptotic and cell cycle arrest genes through regulating activities or expression of DNMTs and HDACs that modify cancer’s epigenetic signature [11,34]. However, there appears to be a dearth of studies investigating the role of sulforaphane as an epigenetic modulator in brain cancer.

Rapha Myr^®^ induces the upregulation of DNA methyltransferase1 (DNMT1) mRNA level and global DNA hypermethylation, unlike the results from other authors reporting that SFN induces DNA hypomethylation and decreased levels of DNMT1 in several types of cancer cells (breast, prostate, colon and liver) [55]. Among epigenetic markers, the sirtuins, a family of NAD^+^ −dependent histone deacetylases comprising seven proteins from Sirt1 to Sirt7, are involved in many crucial cellular processes [56,57]. Their role in cancer is dual depending on the type of tumor, stage and microenvironment. Notably, sirtuins promote migration, growth, epithelial–mesenchymal transition (EMT) and metastasis [57,58]. We demonstrated that Rapha Myr^®^ influences most of the sirtuins except the mitochondrial SIRT3, considered an EMT-repressor and tumor-suppressor protein principally regulating oxidative response, energetic balance, and cellular metabolism, thus promoting genomic and mitochondrial DNA instability. Although the involvement of Sirt 5 in cancer has not yet been investigated, we found out Rapha Myr^®^ modulates its expression, reducing it at its highest concentration [59].

As regards nuclear sirtuins, both Sirt6 and Sirt 7 are considered well-established tumor suppressors and when they are upregulated, they induce apoptosis and cell cycle arrest through HIF1/2 modulation [58,60]. Moreover, Sirt7 is an EMT-promoter. Unlike the literature data, our extract reduces both Sirt6 and Sirt7 expression in astrocytoma cells. Sirt1 instead plays a dual role in tumorigenesis and EMT. Numerous studies report Sirt1 as tumor promoter in several malignant cancers such as breast, colon and prostate cancer. Moreover, SIRT1 also acts as a tumor suppressor directly or by repressing other oncogenes (e.g., Myc). Consistent with this, some tumors including glioma, bladder and ovarian cancer show a low level of SIRT1 [59]. In the context of EMT, SIRT1 has an ambivalent role both as EMT-promoter and EMT-suppressor. Sirt1 increases cellular resistance to apoptosis, senescence, and anoikis and promotes migration and invasion, for instance in both gastric and gastroesophageal junction cancer. On the other hand, Sirt1 suppresses migration and invasion in oral squamous cell carcinoma, lung and ovarian cancer [58].

As regards the nuclear Sirt1, we only observed an increase in the phosphorylated form expression and in the ratio between pSirt1/Sirt1 as well (Figure 9G,H); this data is supported by a moderately significant increase in nuclear activity (Figure 9I). Studies point out that SIRT1 phosphorylation played an important role in regulating the activity of SIRT1 deacetylation, which is responsible for most nuclear deacetylation activity inside the cell [59,61].

All together, the results on epigenoma, stimulating future research direction, suggest Rapha Myr^®^ extract as an epi-drug that could be utilized in human astrocytoma as an integrated therapeutic compound able to modulate cancer development through epigenetic regulation mainly mediated by sirtuins.

## 4. Materials and Methods

### 4.1. Materials

All solvents, chemicals and reference compounds were purchased from Sigma-Aldrich (St. Louis, MO, USA) except as otherwise specified.

### 4.2. Methods

#### 4.2.1. Preparation of Rapha Myr^®^ Extract

Rapha Myr^®^ (Farmabarocco, Ragusa, Italy) was dissolved in water at room temperature (10 mg/mL). Then, the sterile filtered solution was used at different concentrations to treat cell cultures depending on each cell-based assay protocol. Vehicle (water)-treated sample was used as control.

#### 4.2.2. Determination of Antioxidant Activity

The antioxidant activity of Rapha Myr^®^ extract was evaluated by DPPH assay [62]. The reaction mixture contained DPPH radical (86 μM) and different amounts of Rapha Myr^®^ extract (0.5–1.25–2.5–5-10–15% *v/v*) in 1 mL of ethanol. The samples were incubated for 10 min at room temperature, then the absorbance was measured at λ 517 nm with a microplate spectrophotometer reader (Synergy HT multi-mode microplate reader, BioTek, Milano, Italy). The results were expressed as a percentage decrease in absorbance with respect to control.

#### 4.2.3. Cell Cultures

Human astrocytoma cells (1321N1, Sigma-Aldrich, St. Louis, MO, USA) and human glioblastoma cells (U87) from Prof Copani Laboratory (University of Catania) were cultured with MEM (Gibco-BRL Life Technologies, Grand Island, NY, USA), supplemented with 10% fetal bovine serum, 4 mM *L*-glutamine and (50 IU/mL) penicillin/(50 μg/mL) streptomycin.

Human neuroblastoma (SH-SY5Y, ATCC^®^ CRL-2266™, Manassas, VA, USA) cells were cultivated for no more than 20 passages in full medium, i.e., DMEM/F12 supplemented with 10% FBS, 2 mM *L*-glutamine and 100 μg mL^−1^ streptomycin.

Human Foreskin Fibroblast cells (HFF1, ATCC^®^ SCRC-1041™), used as a human model for preliminary toxicity screening, were cultured in Dulbecco’s modified Eagle’s medium [DMEM, 4.5 g/L glucose, penicillin/streptomycin (100 U/mL penicillin and 100 μg/mL streptomycin)] with 15% fetal bovine serum.

As regards p53 gene status, U87, SH-SY5Y and HFF1 cells express the p53 wild type isoform while 1321N1 cells express mutated p53 R213Q.

#### 4.2.4. Cell Morphological Analysis

1321N1, U87, SHSY5Y and HFF1 cells were seeded in 12-well plates at 1.5 × 10^5^ cells per well and treated with Rapha Myr^®^ extract (0.5–1.25–2.5% *v/v*) for 24 h. The morphological changes were observed and the images were captured under an inverted light microscope (EVOS FL imaging systems, AMG Thermo Fisher).

#### 4.2.5. MTT Assay

The potential decrease in cell viability of Rapha Myr^®^ extract was evaluated by MTT, a colorimetric method which measures the reduction in MTT, a yellow tetrazolium salt, to a purple formazan by the mitochondrial dehydrogenase enzyme of living cells [63].

1321N1, U87, SHSY5Y and HFF1 cells (1 × 10^4^ cells/well) were treated with different concentrations of Rapha Myr^®^ extract (0.5–1.25–2.5–5–10% *v/v*) and with water (untreated control) for 24 h; then 200 μL of MTT (0.5 mg/mL) in culture medium were added to each well and incubated for 3 h at 37 °C in a humidified atmosphere with 5% CO_2_. The optical density (OD) was measured with a microplate spectrophotometer reader (Synergy HT multi-mode microplate reader, BioTek, Milano, Italy) at λ 550 nm. The results were expressed as the percentage of cell viability in comparison with untreated control viable cells, whose value was equal to 100%. The IC50 was defined as the concentration that resulted in a 50% decrease in absorbance in treated cells compared to that in untreated cells.

#### 4.2.6. ROS Determination (DCFDA assay)

1321N1 cells were plated in 12-well plates (about 250,000 cells per well) and incubated at 37 °C in a humidified atmosphere with 5% CO_2_. After 24 h, the cells were treated with different concentrations of Rapha Myr^®^ extract (0.5 and 1.25% *v/v*) for 24 h. ROS levels were evaluated on untreated and treated cells using 2′,7′-dichlorofluorescein diacetate [64]. The fluorescence was measured spectrofluorometrically with a microplate reader (Synergy HT multi-mode microplate reader, BioTek, Milano, Italy) λ_excitation_ = 488 nm and λ_emission_ = 525 nm. The total protein content was evaluated for each sample according to Bradford (1976). The results were expressed as fluorescence intensity per mg protein and compared with the control cells.

#### 4.2.7. Total Thiol Groups Determination (DTNB Method)

1321N1 cells were plated in 12-well plates (about 250,000 cells per well) and incubated at 37 °C in a humidified atmosphere with 5% CO_2_. After 24 h, the cells were treated with different concentrations of Rapha Myr^®^ extract (0.5 and 1.25% *v/v*) for 24 h. Total thiol groups, containing predominantly reduced glutathione (GSH), were determined spectrophotometrically at λ 412 nm using 5,5′-dithiobis (2-nitrobenzoic acid) [65]. The total protein content present in the lysates was evaluated for each sample according to Bradford (1976). The results were expressed as nmol GSH/mg proteins calculated referring to a glutathione calibration curve.

#### 4.2.8. Alkaline Comet Assay

Alkaline comet assay was performed on 1321N1 cells untreated and treated with different concentrations of Rapha Myr^®^ extract (0.5 and 1.25% *v/v*) for 24 h, as reported by Tomasello et al., 2017 [66]. After treatments, cells embedded with 0.7% low melting point agarose (LMA) were spotted on pre-treated Flare^TM^ slide (Trevigen Inc., Gaithersburg, MD, USA) and incubated in cold lysis solution (NaCl 2.5 M, Na_2_EDTA 100 mM, TRIS-HCl 8 mM, TRITON 1%, DMSO 10%, pH 10) for 1h at 4 °C. The nucleoids were electrophoresed for 20 min at 0.7 V/cm in alkaline solution (1 mM Na_2_EDTA. 300 mM NaOH), washed with neutralization buffer, dried with 70% ethanol and stained with SYBR Green (1:10,000). Fifty nucleoids were analyzed for each sample using an epifluorescence microscope (Leica, Wetzlar, Germany) equipped with a camera. CASP (1.2.2, CASPLab, University of Wroclaw, Poland) image analysis software was used to evaluate DNA damage. The results were expressed as the percentage of fragmented DNA present in the comet tail (%TDNA).

#### 4.2.9. Wound Healing Assay

The effect of Rapha Myr^®^ extract on 1321N1 cytotoxicity was determined in vitro by Wound Healing assay. Confluent monolayers of 1321N1 cells in 6-well plates were wounded with a pipette tip and washed with PBS 1X to remove detached cells. The wounded monolayers were treated with different concentrations of Rapha Myr^®^ extract (0.5 and 1.25% *v/v*) in starving medium for 24, 48 and 72 h. Wound closure was quantified by measuring the remaining uncovered area with the ImageJ software program (Version 1.43; Broken Symmetry Software, Bethesda, MD, USA) [67].

#### 4.2.10. Immunofluorescence (IF) Detection of Cytoskeleton Proteins

The analysis of cytoskeleton proteins was performed as previously described by Malfa et al. (2014) [68]. Both indirect and direct immunofluorescence was carried out on label α-tubulin and microfilaments, respectively. Samples, dried and mounted with mounting medium (Calbiochem, La Jolla, CA, USA), were visualized with a Confocal Laser Scanning microscope Leica TCS SP8 (AOBS).

#### 4.2.11. Double Indirect Immunofluorescence (IF) Detection of Integrin α5 and Microfilaments on ECM

Cell-free Extracellular matrices were prepared using human dental pulp stem cells (DPSCs) according to S. Laudani et al., 2020 [69].

1321N1 cells (3 × 104) were seeded on extracellular matrix derived from DPSCs, treated with Rapha Myr^®^ (2.5 *v/v*) for 72 h and, after this time, we performed a double indirect IF. Briefly, after blocking with 5% BSA in PBS, primary antibody polyclonal anti Integrin α5 subunit (Cytoplasmatic) (Immunological Science, Rome, Italy) was added (1:1000) to each sample followed by fluorescent secondary antibody Goat anti-rabbit IgG-Alexa Fluor 594 (2.5 μg/mL) (Immunological Sciences, Rome, Italy). Finally, samples were incubated with FITC-Phalloidin (20 μL/mL PBS) for 30 min at 37 °C. After two washes in PBS, samples were allowed to dry and mounted using Fluoro Gel with DAPI (EMS, Hatfield, PA, USA). The immunofluorescence was evaluated with a fluorescence microscope Olympus BX50 equipped with a DC500 camera (Leica).

#### 4.2.12. Cell Cycle Analysis

Cell cycle analysis was performed on untreated and 24 h treated cells with Rapha Myr^®^ extract (0.5–1.25–2.5% *v/v*), according to Malfa et al., 2019 [70]. In order to assess cell cycle state, 1 × 10^6^ cells were stained with propidium iodide (PI, 50 ng/mL) and analyzed by the FlowSight^®^ (Amnis^®^, part of EMD Millipore) imaging flow cytometer. The cell distribution in each cell-cycle phase was determined by using IDEAS 6.0 image analysis software (Amnis Inc., Seattle, WA, USA).

#### 4.2.13. Determination of Apoptosis by Annexin V/PI Staining

The discrimination among live, necrotic, and early and late apoptotic cells, was performed by propidium iodide (PI)/fluorescein-labelled Annexin V staining (AnV-FITC) according to the manufacturer’s instructions (Annexin V-FITC kit, eBioscience, Vienna, Austria) with slight modification. 1321N1 cells were seeded in 60 mm dish at 1.5 × 10^6^ cells/dish and treated with Rapha Myr^®^ extract (0.5–1.25–2.5% *v/v*) for 24 h. After this period, the floating cells were collected and the adherent cells were trypsinized, and both viable and death cells were centrifuged at 100× *g*. Pellets (1 × 10^6^ cells) were suspended in assay buffer, added with Annexin V-fluorescein isothiocyanate (FITC), slightly mixed and incubated for 10 min at room temperature in the dark. Prior to analysis, the samples were stained with PI (20 μg/mL) and 10,000 events were immediately collected by the imaging flow cytometer FlowSight^®^ (Amnis^®^, part of EMD Millipore) and then analysed by using IDEAS 6.0 image analysis software (Amnis Inc., Seattle, WA, USA) [71].

#### 4.2.14. Western Blot Analysis

Whole cell protein of untreated and 24 h Rapha Myr^®^ extract (0.5–1.25–2.5% *v/v*)-treated cells were prepared according to Laemmli (1970) and submitted to Western blot analysis, performed according to Grabowska et al., 2016 [72]. The primary antibodies used were: anti-integrin α5 (1:1000) (Immunological Sciences, Rome, Italy), anti-GAPDH (1:50,000) (Millipore, Darmstadt, Germany), anti-Poly (ADP-ribose)polymerase (PARP, 1:1000) (BD Biosciences, San Jose, CA, USA), anti-γH2AX Ser139 (1:1000) (Abcam, Cambridge, UK), anti-p53 (1:500) (Santa Cruz Biotechnology, Santa Cruz, CA, USA), anti-p21 (1:500) (Sigma-Aldrich, St. Louis, MO, USA), anti-phospho-p53 Ser15 (1:250), anti-sirt 1 (1:250), anti-phospho-sirt 1 Ser47 (1:250), anti-sirt 3 (1:500), anti-sirt 5 (1:500), anti-sirt 6 (1:1000) and anti-sirt 7 (1:250) (Cell Signalling Technology, Denvers, CO, USA). Each protein target was detected by using specific secondary horseradish peroxidase-conjugated antibodies (1:2000) (Dako, Glostrup, Denmark) and an ECL system (Thermo Scientific, Rockford, IL, USA). The expression level of proteins was measured by densitometric analysis using the software Image J and GAPDH was chosen as the reference protein.

#### 4.2.15. Methy-Sens Comet Assay

Methy-sens comet assay was performed on 1321N1 cells untreated and treated with different concentrations of Rapha Myr^®^ extract (0.5 and 1.25% *v/v*) for 24 h, as reported by Perrotti et al., 2015, with slight modifications [73]. After treatments, the cells were agarose embedded, immersed in lysis solution composed as reported above for alkaline Comet assay and maintained overnight at 4 °C. According to Methy-sens protocol, before electrophoresis, the slides were incubated at 37 °C for 10 min in a humidity chamber with 50 µL of Working HpaII or MspI Enzyme solution (5 µL/mL in FD buffer; Thermo Fisher Scientific, Waltham, MA, USA) spotted on each sample area of the Flare^TM^ slide. Control samples were simultaneously incubated with only 50 µL of FD buffer (control buffer). Both enzymes recognize and cleave the sequence C|CGG in double-stranded DNA, but whereas *MspI* cleaves the DNA-independent of its methylation status, *Hpa*II is unable to cleave when the second cytosine is methylated (C|^me^CGG).

The nucleoids were electrophoresed, dried with 70% ethanol and stained with SYBR Green (1:10,000) as for alkaline comet assay. Fifty nucleoids were analyzed for each sample, and the percentage ratio between % TDNA value of each enzyme treatment [(%TDNA [HpaII]/%TDNA [MspI])*100] was chosen as the parameter to assess global DNA methylation.

#### 4.2.16. RNA Isolation and Real-Time Reverse Transcription-Polymerase Chain Reaction

Total RNA was isolated from 1321N1 cells using TRIzol reagent (Life Technologies, Carlsbad, CA, USA) according to the manufacturer’s instructions. The reverse transcription was carried out on 1 µg of total RNA using the QuantiTect^®^ Reverse Transcription Kit (Qiagen Inc., Valencia, MD, USA) [74]. The DNMT1 mRNA expression level was determined by RT-PCR (QuantiNova SYBR^®^ Green RT-PCR Kit) on Rotor-Gene Q 5PLEX (Qiagen Inc., Valencia, MD, USA). Validated primers were purchased from Qiagen Inc: DNMT1 (QT00034335) and RPL0 (QT00075012). The comparative threshold cycle method (ΔΔ*C*t model) was performed for relative quantification and RPL0 was chosen as the reference gene. The results were expressed as Fold changes (2^−ΔΔ*C*t^) relative to control (set as 1).

#### 4.2.17. Nuclear Sirtuins Activity

Nuclear extracts were obtained from cells untreated and treated with different concentrations of Rapha Myr^®^ extract (0.5 and 1.25% *v/v*) for 24 h by using the ab113474 kit (Abcam, Cambridge, UK). Nuclear sirtuins activity was evaluated by using the Universal SIRT Activity Assay Kit (ab156915, Abcam, Cambridge, UK). The absorbance was measured with a microplate spectrophotometer reader (Synergy HT multi-mode microplate reader, BioTek, Milano, Italy) at λ 450 nm. The activity for each sample was expressed as OD/min/mg proteins and was calculated according to manufacturer’s protocol [75].

#### 4.2.18. Statistical Analysis

All the results were obtained by three independent experiments each performed in triplicate and expressed as mean ± standard deviation (SD). Statistical differences among different treatments were assessed by one-way ANOVA. Post hoc comparison was performed according to the Bonferroni test. We applied *p* < 0.05 as the minimum level of significance. All the analyses were performed using Graph Prism version 5.

## 5. Conclusions

SFN has been recognized as a safe agent for its low toxicity towards normal cells; it can rapidly cross the blood brain barrier and accumulate in the CNS after administration and can overcome the chemoresistance of tumor cells [3]. Our work provides additional insight into chemotherapeutic capability of SFN showing the effects elicited by Rapha Myr^®^, a broccoli seed extract plus active myrosinase, on human astrocytoma cells with mutant p53 R213Q. Rapha Myr^®^ showed antiproliferative and cytotoxic effects exerted by modulating some epigenetic pathways and inducing significant alteration of cytoarchitecture morphology. This data, together with the induction of apoptosis, inhibition of α5 translocation and low adhesion on ECM, reduction in cell migration and integrin α5 expression, allowed us to suggest a contribution of the mixture in restoring anoikis sensitivity in human astrocytoma cells through epigenetic regulation and the functional reduction in the p53 oncogenic isoform responsible for the aggressive and invasive nature of this tumor. Rapha Myr^®^, due to the mix of sulforaphane and myrosinase, has been proven to be a particularly efficient natural supplement, which could be ascribed among the nutraceuticals. Besides the necessity of further in vivo and in vitro studies to fully elucidate the chemopreventive/chemotherapeutic properties of SFN and other isothiocyanates, we surely can point out Rapha Myr^®^ as a valuable compound for integrated oncology strategies in the prevention and treatment of human astrocytoma.

## Supplementary Materials

Supplementary Materials can be found at https://www.mdpi.com/1422-0067/21/15/5328/s1. Figure S1: Cell morphology of SHSY5Y (neuroblastoma cells) untreated and treated for 24 h with different concentrations of Rapha Myr^®^. Images were acquired by optical inverted light microscopy, original magnification 10×; Figure S2: Cell morphology of U87 (glioblastoma cells) untreated and treated for 24 h with different concentrations of Rapha Myr^®^. Images were acquired by optical inverted light microscopy, original magnification 10×; Figure S3: Cell morphology of HFF1 (fibroblast cells) untreated and treated for 24 h with different concentrations of Rapha Myr^®^. Images were acquired by optical inverted light microscopy, original magnification 10×; Figure S4: Abnormal mitotic spindles. The merge was made between spindle microtubules and DAPI-stained chromosomes. Scale bar: 20 μm; Figure S5: Nuclear morphology (DAPI) and percentages values of normal, small (s), misshappen or fragmented (m) nuclei in 1321N1 cells untreated and treated with of 0.5% and 1.25% *v/v* Rapha Myr^®^ extract for 72 h; Figure S6: Representative flow cytometry cell cycle histogram. Cell cycle analysis by flow cytometry G1, S, and G2/M phase in human astrocytoma (1321N1) cells treated with different concentrations of Rapha Myr^®^ for 24 h. For the graph see main manuscript (Figure 8A); Figure S7: Representative flow cytometry scatter plots showing apoptosis of human astrocytoma (1321N1) cells stained with annexin V-fluorescein isothiocyanate (FITC)/propidium iodide following treatment with different concentrations of Rapha Myr^®^ for 24 h. For the graph see main manuscript (Figure 8B).

## Abbreviations

ITCs	Isothiocyanates
SFN	Sulforaphane
GR	Glucoraphanin
GSH	Reduced Glutathione
DNA double-strand breaks	DSBs
Sirt	Sirtuin
EMT	Epithelial-Mesenchymal Transition
ECM	Extracellular matrix
IF	Immunofluorescence

## References

[1] Loomans-Kropp H.A., Umar A. (2019). Cancer prevention and screening: The next step in the era of precision medicine. npj Precis. Oncol..

[2] Palumbo M.O., Kavan P., Miller W.H., Panasci L., Assouline S., Johnson N., Cohen V., Patenaude F., Pollak M., Jagoe R.T. (2013). Systemic cancer therapy: Achievements and challenges that lie ahead. Front. Pharmacol..

[3] Sita G., Hrelia P., Graziosi A., Morroni F. (2018). Sulforaphane from Cruciferous Vegetables: Recent Advances to Improve Glioblastoma Treatment. Nutrients.

[4] Block K.I., Gyllenhaal C., Lowe L., Amedei A., Amin A.R.M.R., Amin A., Aquilano K., Arbiser J., Arreola A., Arzumanyan A. (2015). Designing a broad-spectrum integrated approach for cancer prevention and treatment. Semin. Cancer Biol..

[5] Guadamillas M.C., Cerezo A., del Pozo M.A. (2011). Overcoming anoikis-pathways to anchorage-independent growth in cancer. J. Cell Sci..

[6] Paoli P., Giannoni E., Chiarugi P. (2013). Anoikis molecular pathways and its role in cancer progression. Biochim. Biophys. Acta.

[7] Stupack D.G., Teitz T., Potter M.D., Mikolon D., Houghton P.J., Kidd V.J., Lahti J.M., Cheresh D.A. (2006). Potentiation of neuroblastoma metastasis by loss of caspase-8. Nature.

[8] Flavahan W.A., Gaskell E., Bernstein B.E. (2017). Epigenetic plasticity and the hallmarks of cancer. Science.

[9] Kanwala R., Gupta S. (2012). Epigenetic modifications in cancer. Clin. Genet..

[10] Falzone L., Romano G.L., Salemi R., Bucolo C., Tomasello B., Lupo G., Anfuso C.A., Spandidos D.A., Libra M., Candido S. (2019). Prognostic significance of deregulated microRNAs in uveal melanomas. Mol. Med. Rep..

[11] Carlos-Reyes Á., López-González J.S., Meneses-Flores M., Gallardo-Rincón D., Ruíz-García E., Marchat L.A., Astudillo-de la Vega H., Hernández de la Cruz O.N., López-Camarillo C. (2019). Dietary Compounds as Epigenetic Modulating Agents in Cancer. Front. Genet..

[12] Shukla S., Penta D., Mondal P., Meeran S.M. (2019). Epigenetics of Breast Cancer: Clinical Status of Epi-drugs and Phytochemicals. Adv. Exp. Med. Biol..

[13] Ranjan A., Ramachandran S., Gupta N., Kaushik I., Wright S., Srivastava S., Das H., Srivastava S., Prasad S., Srivastava S.K. (2019). Role of Phytochemicals in Cancer Prevention. Int. J. Mol. Sci..

[14] Wu X., Zhou Q.H., Xu K. (2009). Are isothiocyanates potential anti-cancer drugs?. Acta Pharmacol. Sin..

[15] Bayat Mokhtari R., Baluch N., Homayouni T.S., Morgatskaya E., Kumar S., Kazemi P., Yeger H. (2018). The role of Sulforaphane in cancer chemoprevention and health benefits: A mini-review. J. Cell Commun. Signal..

[16] Kaufman-Szymczyk A., Majewski G., Lubecka-Pietruszewska K., Fabianowska-Majewska K. (2015). The Role of Sulforaphane in Epigenetic Mechanisms, Including Interdependence between Histone Modification and DNA Methylation. Int. J. Mol. Sci..

[17] Liu P., Atkinson S.J., Akbareian S.E., Zhou Z., Munsterberg A., Robinson S.D., Bao Y. (2017). Sulforaphane exerts anti-angiogenesis effects against hepatocellular carcinoma through inhibition of STAT3/HIF-1α/VEGF signalling. Sci. Rep..

[18] Kensler T.W., Egner P.A., Agyeman A.S., Visvanathan K., Groopman J.D., Chen J.G., Chen T.Y., Fahey J.W., Talalay P. (2013). Keap1-nrf2 signaling: A target for cancer prevention by sulforaphane. Top. Curr. Chem..

[19] Lewinska A., Adamczyk-Grochala J., Deregowska A., Wnuk M. (2017). Sulforaphane-Induced Cell Cycle Arrest and Senescence are accompanied by DNA Hypomethylation and Changes in microRNA Profile in Breast Cancer Cells. Theranostics.

[20] Burnett J.P., Lim G., Li Y., Shah R.B., Lim R., Paholak H.J., McDermott S.P., Sun L., Tsume Y., Bai S. (2017). Sulforaphane enhances the anticancer activity of taxanes against triple negative breast cancer by killing cancer stem cells. Cancer Lett..

[21] Lubecka-Pietruszewska K., Kaufman-Szymczyk A., Stefanska B., Cebula-Obrzut B., Smolewski P., Fabianowska-Majewska K. (2015). Sulforaphane Alone and in Combination with Clofarabine Epigenetically Regulates the Expression of DNA Methylation-Silenced Tumour Suppressor Genes in Human Breast Cancer Cells. J. Nutr. Nutr..

[22] Tsai J.Y., Tsai S.H., Wu C.C. (2019). The chemopreventive isothiocyanate sulforaphane reduces anoikis resistance and anchorage-independent growth in non-small cell human lung cancer cells. Toxicol. Appl. Pharmacol..

[23] Pereira L.P., Silva P., Duarte M., Rodrigues L., Duarte C.M., Albuquerque C., Serra A.T. (2017). Targeting Colorectal Cancer Proliferation, Stemness and Metastatic Potential Using Brassicaceae Extracts Enriched in Isothiocyanates: A 3D Cell Model-Based Study. Nutrients.

[24] Ming Y., Meng R., Yue Q., Wendi T., Zhongpeng W., Hao L., Qipeng Y. (2016). Sulforaphene inhibits hepatocellular carcinoma through repressing keratin 8 and activating anoikis. RSC Adv..

[25] Fahey J.W., Wade K.L., Stephenson K.K., Panjwani A.A., Liu H., Cornblatt G., Cornblatt B.S., Ownby S.L., Fuchs E., Holtzclaw W.D. (2019). Bioavailability of Sulforaphane Following Ingestion of Glucoraphanin-Rich Broccoli Sprout and Seed Extracts with Active Myrosinase: A Pilot Study of the Effects of Proton Pump Inhibitor Administration. Nutrients.

[26] Curran K.M., Bracha S., Wong C.P., Beaver L.M., Stevens G.F., Ho E. (2018). Sulforaphane absorption and histone deacetylase activity following single dosing of broccoli sprout supplement in normal dogs. Vet. Med. Sci..

[27] Fahey J.W., Wehage S.L., Holtzclaw W.D., Kensler T.W., Egner P.A., Shapiro T.A., Talalay P. (2012). Protection of humans by plant glucosinolates: Efficiency of conversion of glucosinolates to isothiocyanates by the gastrointestinal microflora. Cancer Prev. Res..

[28] Clarke J.D., Hsu A., Riedl K., Bella D., Schwartz S.J., Stevens J.F., Ho E. (2011). Bioavailability and inter-conversion of sulforaphane and erucin in human subjects consuming broccoli sprouts or broccoli supplement in a cross-over study design. Pharmacol. Res..

[29] Rutka J.T., Akiyama Y., Lee S.P., Ivanchuk S., Tsugu A., Hamel P.A. (2000). Alterations of the p53 and pRB Pathways in Human Astrocytoma. Rev. Brain Tumor Pathol..

[30] Kalia M. (2015). Biomarkers for personalized oncology: Recent advances and future challenges. Metabolism.

[31] Cirrone G.A.P., Margarone D., Maggiore M., Anzalone A., Borghesi M., Jia S.B., Bulanov S.S., Bulanov S., Carpinelli M., Cavallaro S. (2013). ELIMED: A New Hadron Therapy Concept Based on Laser Driven Ion Beams. Proceedings of the SPIE Optics + Optoelectronics.

[32] Zhong X., Rescorla F.J. (2012). Cell surface adhesion molecules and adhesion-initiated signaling: Understanding of anoikis resistance mechanisms and therapeutic opportunities. Cell Signal..

[33] Mentella M.C., Scaldaferri F., Ricci C., Gasbarrini A., Miggiano G.A.D. (2019). Cancer and Mediterranean Diet: A Review. Nutrients.

[34] Paul B., Li Y., Tollefsbol T.O. (2018). The Effects of Combinatorial Genistein and Sulforaphane in Breast Tumor Inhibition: Role in Epigenetic Regulation. Int. J. Mol. Sci..

[35] Azarenko O., Okouneva T., Singletary K.W., Jordan M.A., Wilson L. (2008). Suppression of microtubule dynamic instability and turnover in MCF7 breast cancer cells by sulforaphane. Carcinogenesis.

[36] Lockett S., Verma C., Brafman A., Gudla P., Nandy K., Mimaki Y., Fuchs P.L., Jaja J., Reilly K.M., Beutler J. (2014). Quantitative Analysis of F-Actin Redistribution in Astrocytoma Cells Treated with Candidate Pharmaceuticals. Cytom. Part A.

[37] Byun S., Shin S.H., Park J., Lim S., Lee E., Lee C., Sung D., Farrand L., Lee S.R., Kim K.H. (2016). Sulforaphene suppresses growth of colon cancer-derived tumors via induction of glutathione depletion and microtubule depolymerization. Mol. Nutr. Food Res..

[38] Malric L., Monferran S., Gilhodes J., Boyrie S., Dahan P., Skuli N., Sesen J., Filleron T., Kowalski-Chauvel A., Cohen-Jonathan Moyal E. (2017). Interest of integrins targeting in glioblastoma according to tumor heterogeneity and cancer stem cell paradigm: An update. Oncotarget.

[39] Renner G., Noulet F., Mercier M.C., Choulier L., Etienne- Selloum N., Gies J.P., Lehmann M., Lelong-Rebel I., Martin S., Dontenwill M. (2016). Expression/activation of alpha5beta1 integrin is linked to the beta-catenin signaling pathway to drive migration in glioma cells. Oncotarget.

[40] Maglott A., Bartik P., Cosgun S., Klotz P., Ronde P., Fuhrmann G., Takeda K., Martin S., Dontenwill M. (2006). The small alpha5beta1 integrin antagonist, SJ749, reduces proliferation and clonogenicity of human astrocytoma cells. Cancer Res..

[41] Pankov R., Cukierman E., Katz B.Z., Matsumoto K., Lin D.C., Lin S., Hahn C., Yamada K.M. (2000). Integrin Dynamics and Matrix Assembly: Tensin-dependent Translocation of α5β1 Integrins Promotes Early Fibronectin Fibrillogenesis. J. Cell Biol..

[42] Redon C.E., Nakamura A.J., Zhang Y.W., Ji J.J., Bonner W.M., Kinders R.J., Parchment R.E., Doroshow J.H., Pommier Y. (2010). Histone gammaH2AX and poly(ADP-ribose) as clinical pharmacodynamic biomarkers. Clin. Cancer Res..

[43] Sekine-Suzuki E., Yu D., Kubota N., Okayasu R., Anzai K. (2008). Sulforaphane induces DNA double strand breaks predominantly repaired by homologous recombination pathway in human cancer cells. Biochem. Biophys. Res. Commun..

[44] Hoffman J.D., Ward W.M., Loo G. (2015). Effect of antioxidants on the genotoxicity of phenethyl isothiocyanate. Mutagenesis.

[45] Żuryń A., Litwiniec A., Safiejko-Mroczka B., Klimaszewska-Wiśniewska A., Gagat M., Krajewski A., Gackowska L., Grzanka D. (2016). The effect of sulforaphane on the cell cycle, apoptosis and expression of cyclin D1 and p21 in the A549 non-small cell lung cancer cell line. Int. J. Oncol..

[46] Clarke J.D., Hsu A., Yu Z., Dashwood R.H., Ho E. (2011). Differential effects of sulforaphane on histone deacetylases, cell cycle arrest and apoptosis in normal prostate cells versus hyperplastic and cancerous prostate cells. Mol. Nutr. Food Res..

[47] Olivier M., Hollstein M., Hainaut P. (2010). TP53 mutations in human cancers: Origins, consequences, and clinical use. Cold Spring Harb. Perspect. Biol..

[48] Pan Y., Haines D.S. (2000). Identification of a tumor-derived p53 mutant with novel transactivating selectivity. Oncogene.

[49] Zhang Y., Zhang Y.J., Zhao H.Y., Zhai Q.L., Zhang Y., Shen Y.F. (2014). The impact of R213 mutation on p53-mediated p21 activity. Biochimie.

[50] Lenzi M., Fimognari C., Hrelia P. (2014). Sulforaphane as a Promising Molecule for Fighting Cancer. Cancer Treat. Res..

[51] Powell E., Piwnica-Worms D., Piwnica-Worms H. (2014). Contribution of p53 to metastasis. Cancer Discov..

[52] El-Deiry W.S. (2016). P21 (WAF1) mediates cell-cycle inhibition, relevant to cancer suppression and therapy. Cancer Res..

[53] Cordani M., Butera G., Pacchiana R., Masetto F., Mullappilly N., Riganti C., Donadelli M. (2020). Mutant p53-Associated Molecular Mechanisms of ROS Regulation in Cancer Cells. Biomolecules.

[54] Naletova I., Satriano C., Curci A., Margiotta N., Natile G., Arena G., La Mendola D., Nicoletti V., Rizzarelli E. (2018). Cytotoxic phenanthroline derivatives alter metallostasis and redox homeostasis in neuroblastoma cells. Oncotarget.

[55] Pop S., Enciu A.M., Tarcomnicu I., Gille E., Tanase C. (2019). Phytochemicals in cancer prevention: Modulating epigenetic alterations of DNA methylation. Phytochem. Rev..

[56] Tomasello B., Malaguarnera M., Renis M., Di Giacomo C. (2020). Physical Exercise and oxidative stress biomarkers in the elderly. Biochim. Clin..

[57] Carafa V., Rotili D., Forgione M., Cuomo F., Serretiello E., Hailu G.S., Jarho E., Lahtela-Kakkonen M., Mai A., Altucci L. (2016). Sirtuin functions and modulation: From chemistry to the clinic. Clin. Epigenet..

[58] Palmirotta R., Cives M., Della-Morte D., Capuani B., Lauro D., Guadagni F., Silvestris F. (2016). Sirtuins and Cancer: Role in the Epithelial-Mesenchymal Transition. Oxid. Med. Cell. Longev..

[59] Carafa V., Altucci L., Nebbioso A. (2019). Dual Tumor Suppressor and Tumor Promoter Action of Sirtuins in Determining Malignant Phenotype. Front. Pharmacol..

[60] Gilkes D.M., Xiang L., Lee S.J., Chaturvedi P., Hubbi M.E., Wirtz D., Semenza G.L. (2014). Hypoxia-inducible factors mediate coordinated RhoA-ROCK1 expression and signaling in breast cancer cells. Proc. Natl. Acad. Sci. USA.

[61] Sasaki T., Maier B., Koclega K.D., Chruszcz M., Gluba W., Stukenberg P.T., Minor W., Scrable H. (2008). Phosphorylation regulates SIRT1 function. PLoS ONE.

[62] Acquaviva R., Genovese C., Amodeo A., Tomasello B., Malfa G., Sorrenti V., Tempera G., Addamo A.P., Ragusa S., Tundis R. (2018). Biological activities of *Teucrium flavum* L., *Teucrium fruticans* L., and *Teucrium siculum* rafin crude extracts. Plant Biosyst. Int. J. Deal. Asp. Plant Biol..

[63] Di Mauro M.D., Tomasello B., Giardina R.C., Dattilo S., Mazzei V., Sinatra F., Caruso M., D’Antona N., Renis M. (2017). Sugar and mineral enriched fraction from olive mill wastewater for promising cosmeceutical application: Characterization, in vitro and in vivo studies. Food Funct..

[64] Acquaviva R., Sorrenti V., Santangelo R., Cardile V., Tomasello B., Malfa G., Vanella L., Amodeo A., Mastrojeni S., Pugliese M. (2016). Effects of extract of *Celtis aetnensis* (Tornab.) Strobl twigs in human colon cancer cell cultures. Oncol. Rep..

[65] Spampinato M., Murabito P., Raffaele M., Vanella L., Licari M., Distefano A., Tomasello B., Sferrazzo G., Carota G., Di Rosa M. (2019). N-Acetylicysteine restores endogenous antioxidant system in human bronchial epithelial cells exposed to cigarette smoke extract. Euro-Mediterr. Biomed. J..

[66] Tomasello B., Malfa G.A., Strazzanti A., Gangi S., Di Giacomo C., Basile F., Renis M. (2017). Effects of physical activity on systemic oxidative/DNA status in breast cancer survivors. Oncol. Lett..

[67] Olivieri M., Cristaldi M., Pezzino S., Lupo G., Anfuso C.D., Gagliano C., Genovese C., Rusciano D. (2018). Experimental Evidence of the Healing Properties of Lactobionic Acid for Ocular Surface Disease. Cornea.

[68] Malfa G.A., Tomasello B., Sinatra F., Villaggio G., Amenta F., Avola R., Renis M. (2014). Reactive response evaluation of primary human astrocytes after methylmercury exposure. J. Neurosci. Res..

[69] Laudàni S., La Cognata V., Iemmolo R., Bonaventura G., Villaggio G., Saccone S., Barcellona M.L., Cavallaro S., Sinatra F. (2020). Effect of a Bone Marrow-Derived Extracellular Matrix on Cell Adhesion and Neural Induction of Dental Pulp Stem Cells. Front. Cell Dev. Biol..

[70] Malfa G.A., Tomasello B., Acquaviva R., Genovese C., La Mantia A., Cammarata F.P., Ragusa M., Renis M., Di Giacomo C. (2019). *Betula etnensis* Raf. (Betulaceae) Extract Induced HO-1 Expression and Ferroptosis Cell Death in Human Colon Cancer Cells. Int. J. Mol. Sci..

[71] Cardullo N., Barresi V., Muccilli V., Spampinato G., D’Amico M., Condorelli D.F., Tringali C. (2020). Synthesis of Bisphenol Neolignans Inspired by Honokiol as Antiproliferative Agents. Molecules.

[72] Grabowska W., Suszek M., Wnuk M., Lewinska A., Wasiak E., Sikora E., Bielak-Zmijewska A. (2016). Curcumin elevates sirtuin level but does not postpone in vitro senescence of human cells building the vasculature. Oncotarget.

[73] Perotti A., Rossi V., Mutti A., Buschini A. (2015). Methy-sens Comet assay and DNMTs transcriptional analysis as a combined approach in epigenotoxicology. Biomarkers.

[74] Tomasello B., Malfa G.A., La Mantia A., Miceli N., Sferrazzo G., Taviano M.F., Di Giacomo C., Renis M., Acquaviva R. (2019). Anti-adipogenic and anti-oxidant effects of a standardised extract of Moro blood oranges (*Citrus sinensis* (L.) Osbeck) during adipocyte differentiation of 3T3-L1 preadipocytes. Nat. Prod. Res..

[75] Park M.H., Gutiérrez-García A.K., Choudhury M. (2019). Mono-(2-ethylhexyl) phthalate aggravates inflammatory response via sirtuin regulation and nflammasome Activation in RAW 264.7 Cells. Chem. Res. Toxicol..

