# Supplementary material for: Rapha Myr®, a Blend of Sulforaphane and Myrosinase, Exerts Antitumor and Anoikis-Sensitizing Effects on Human Astrocytoma Cells Modulating Sirtuins and DNA Methylation
Source: Int J Mol Sci. 2020 Jul 27;21(15):5328. doi: 10.3390/ijms21155328 (PMC7432334; doi:10.3390/ijms21155328)
Supplement: Supplementary file 1 [file ijms-21-05328-s001.pdf]

Article

Rapha Myr®, a Blend of Sulforaphane and Myrosinase, Exerts Antitumor and Anoikis-Sensitizing Effects on Human Astrocytoma Cells Modulating Sirtuins and DNA Methylation

Barbara Tomasello [1,*], Maria Domenica Di Mauro [1], Giuseppe Antonio Malfa [1], Rosaria Acquaviva [1], Fulvia Sinatra [2], Giorgia Spampinato [3], Samuele Laudani [2], Giusy Villaggio [2], Anna Bielak-Zmijewska [4], Wioleta Grabowska [4], Ignazio Alberto Barbagallo [1], Maria Teresa Liuzzo [5], Elisabetta Sbisà [6], Maria Grazia Forte [7], Claudia Di Giacomo [1], Massimo Bonucci [8] and Marcella Renis [1,*]

[1] Department of Drug Science, Section of Biochemistry, University of Catania, Viale A. Doria 6, 95125 Catania, Italy; mariadomenica.dimauro@virgilio.it (M.D.D.M.); g.malfa@unict.it (G.A.M.); racquavi@unict.it (R.A.); ignazio.barbagallo@unict.it (I.A.B.); cdigiac@unict.it (C.D.G.)

[2] Department of Biomedical and Biotechnological Sciences, University of Catania, Via Santa Sofia 87, 95125 Catania, Italy; sinatra@unict.it (F.S.); s.laudani91@hotmail.it (S.L.); giusyvillaggio@gmail.com (G.V.)

[3] Services Center B.R.I.T. of the University of Catania, 95124 Catania, Italy; giorgiaspampinato@unict.it

[4] Nencki Institute of Experimental Biology, Polish Academy of Sciences, 3 Pasteur St, 02-093 Warsaw, Poland: a.bielak@nencki.gov.pl (A.B.-Z.); w.grabowska@nencki.gov.pl (W.G.)

[5] Medical Clinic of Clinical Allergology and Immunology, 97018 Scicli, Italy; mariateresaliuzzo@gmail.com

[6] Institute of Biomedical Technologies -National Research Council Bari, 70126 Bari, Italy; elisabetta.sbisa@ba.itb.cnr.it

[7] Dept. Prevention ASL BA—SIAN Metropolitan Area Bari, 70120 Bari, Italy; mariagrazia.forte@asl.bari.it

[8] Association Research Center for Integrative Oncology Treatments (ARTOI), 00165 Rome, Italy; maxbonucci@artoi.it

* Correspondence: btomase@unict.it (B.T.); renis@unict.it (M.R.); Tel.: +39-095-7384063 (B.T.); Fax: +39-095-7384220 (B.T.)

Figure S1

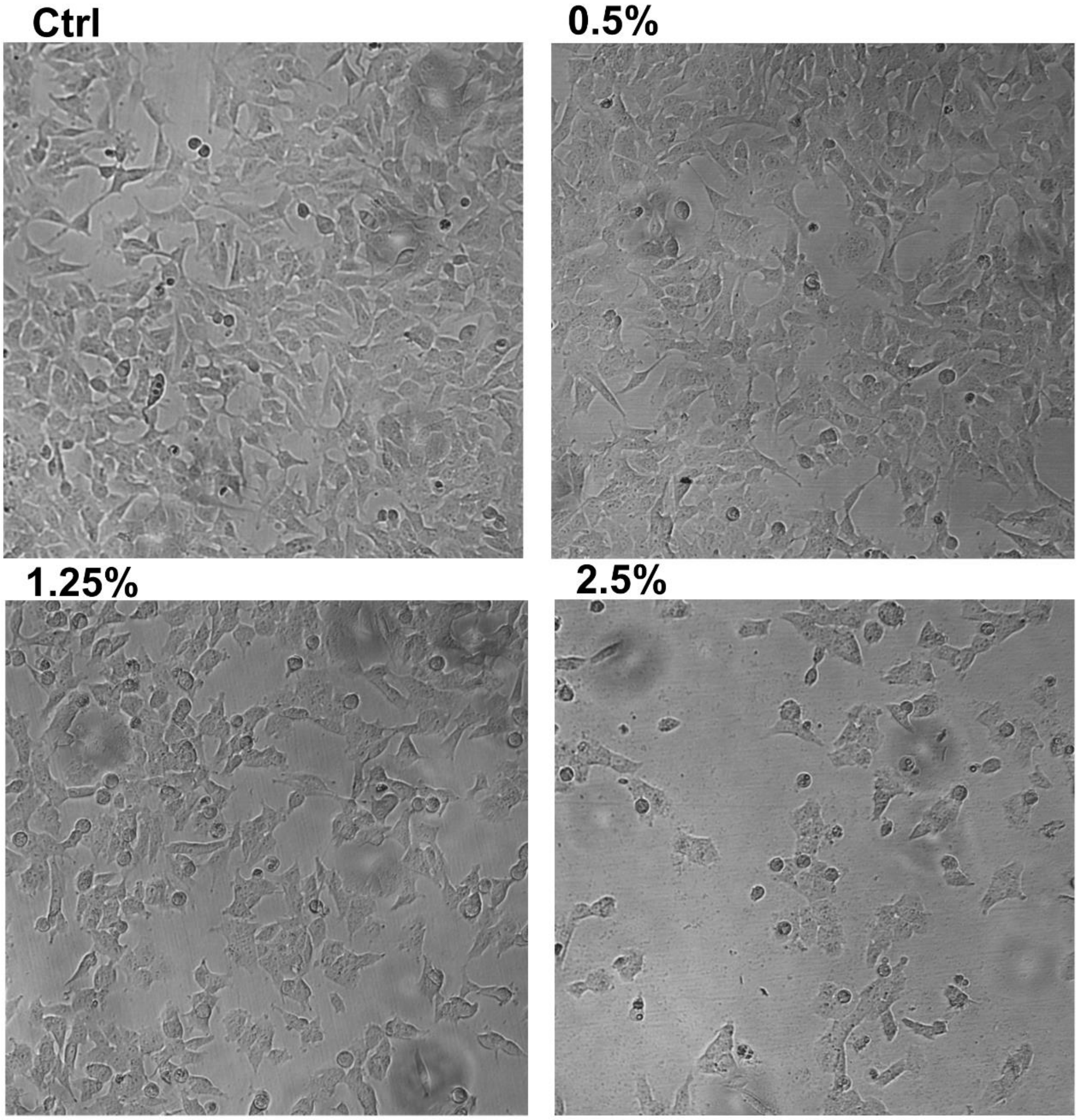

Figure S1: Cell morphology of SHSY5Y (neuroblastoma cells) untreated and treated for 24h with different concentrations of Rapha Myr®. Images were acquired by optical inverted light microscopy, original magnification 10x, scale bare 400μm.

Figure S2

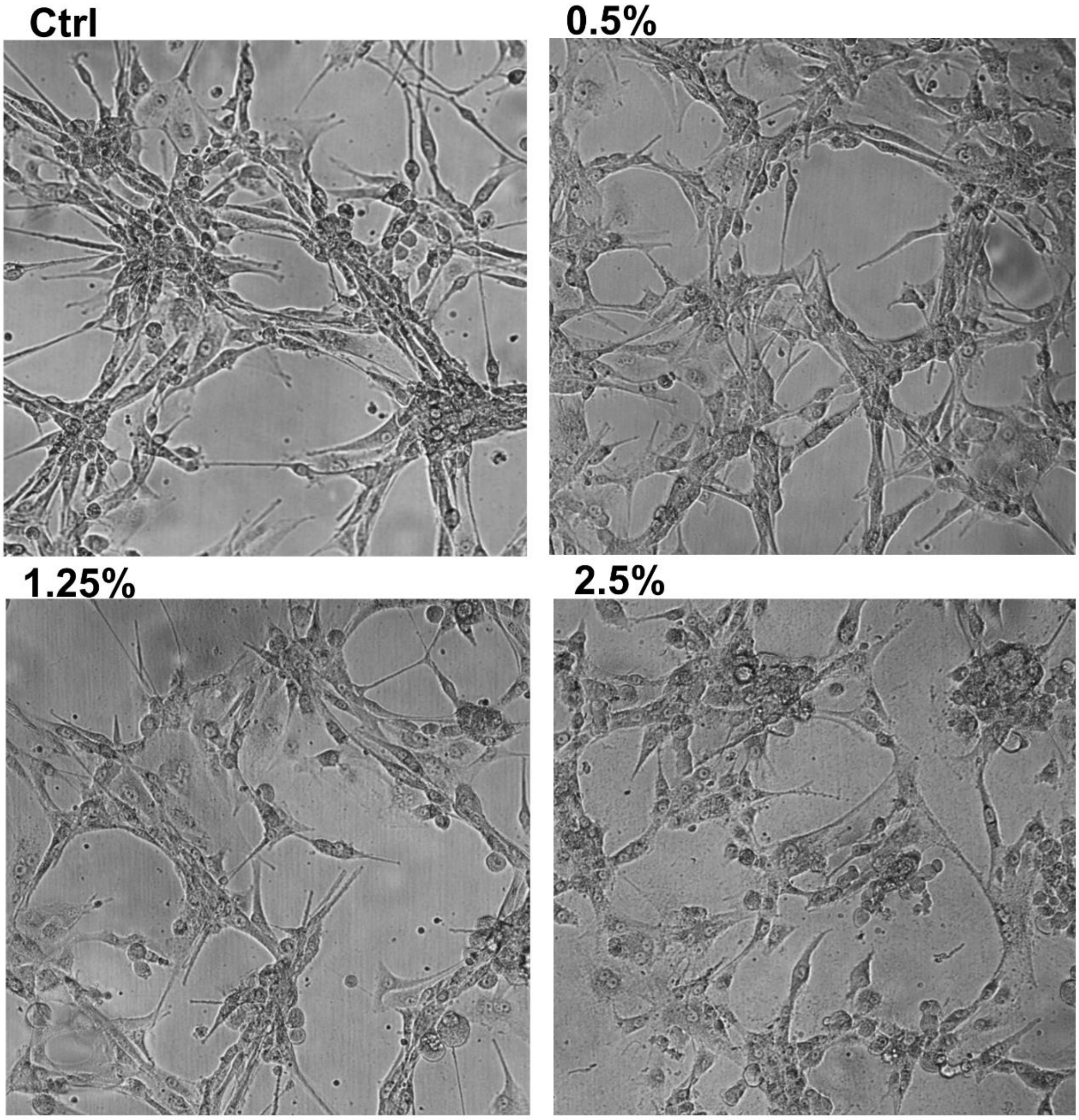

Figure S2: Cell morphology of U87 (glioblastoma cells) untreated and treated for 24h with different concentrations of Rapha Myr®. Images were acquired by optical inverted light microscopy, original magnification 10x, scale bare 400μm.

Figure S3

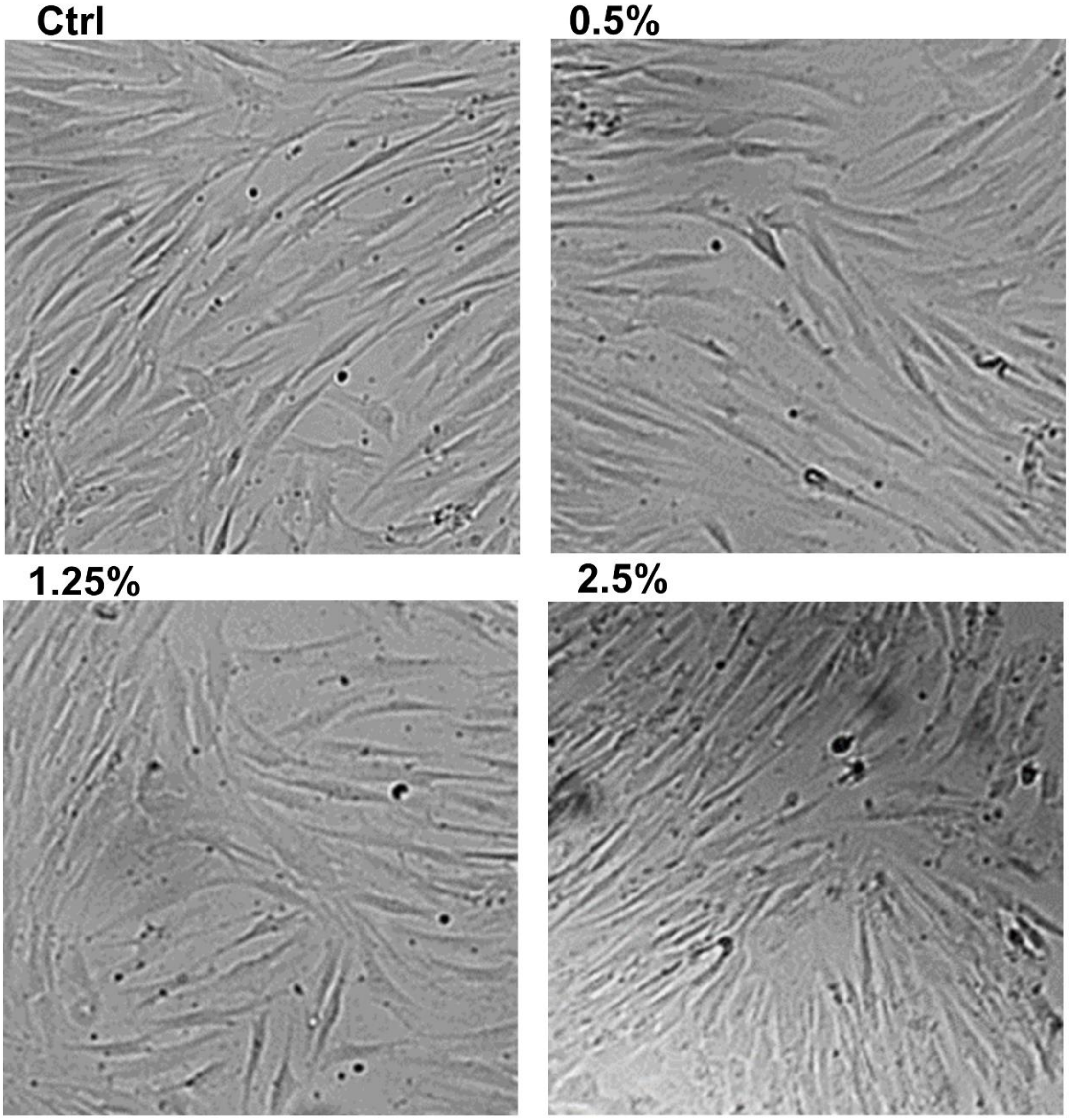

Figure S3: Cell morphology of HFF1 (fibroblast cells) untreated and treated for 24h with different concentrations of Rapha Myr®. Images were acquired by optical inverted light microscopy, original magnification 1010x, scale bare 400μm.

Figure S4

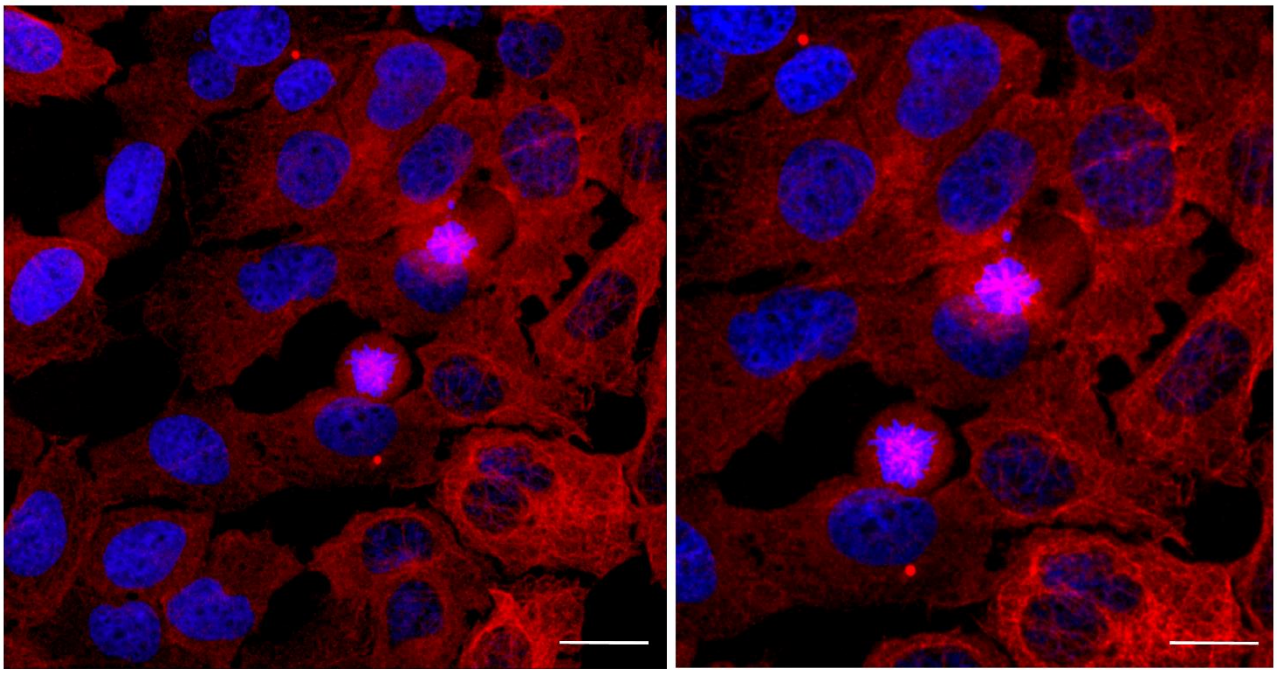

Figure S4: Abnormal mitotic spindles. The merge was made between spindle microtubules and DAPI-stained chromosomes. Scale bar: 20 µm

Figure S5 nuclei quantification

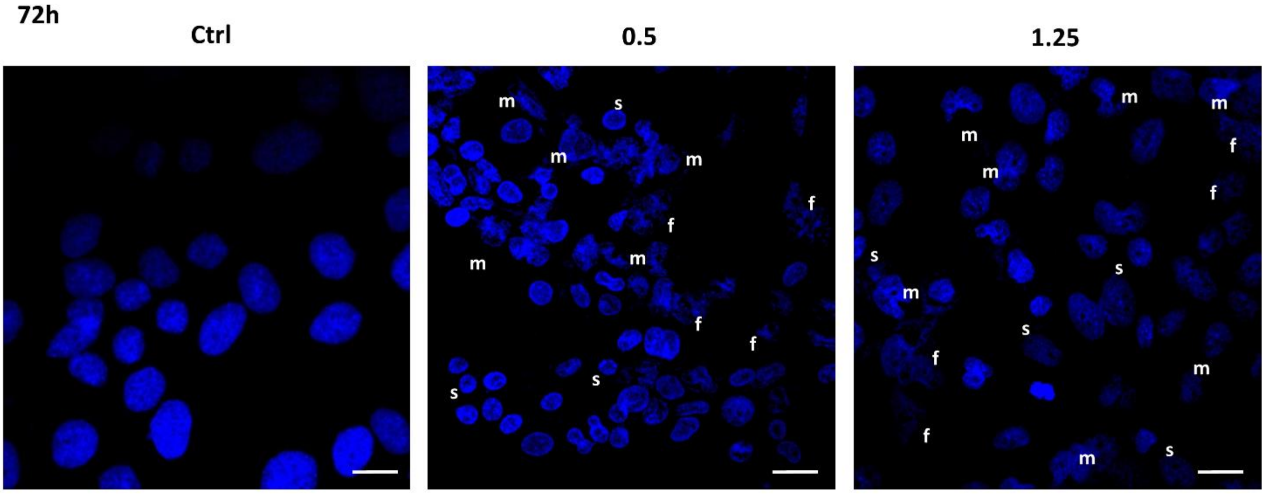

Nuclei	Ctrl	0.5	1.25
normal	97%	10,0%	3,0%
small	3%	74,0%	88,0%
misshapen fragmented	—	16,0%	9,7%

Figure S5: Nuclear morphology (DAPI) and percentages values of normal, small (s), misshapen or fragmented (m) nuclei in 1321N1 cells untreated and treated with of 0.5% and 1.25% v/v Rapha Myr® extract for 72 h.

Figure S6

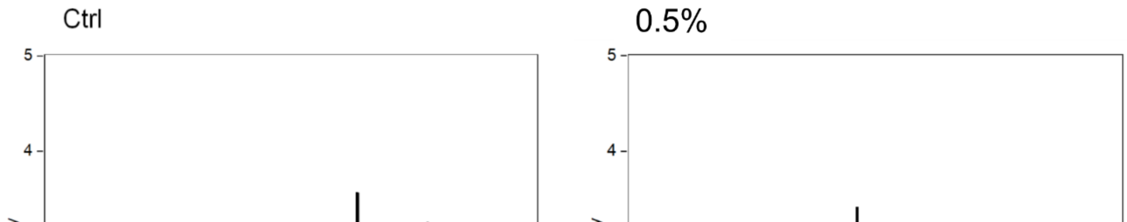

Figure S6: Representative flow cytometry cell cycle histogram. Cell cycle analysis by flow cytometry G1, S, and G2/M phase in human astrocytoma (1321N1) cells treated with different concentrations of Rapha Myr® for 24h. For the graph see main manuscript (Figure 8A).

Figure S7

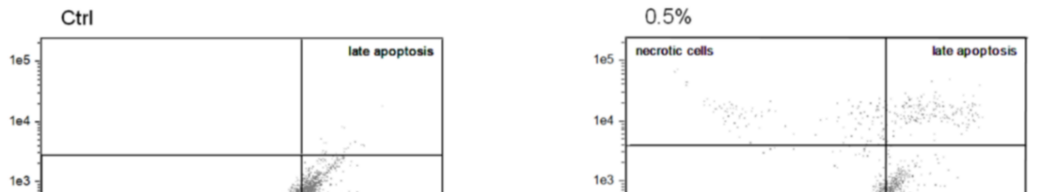

Figure S7: Representative flow cytometry scatter plots showing apoptosis of human astrocytoma (1321N1) cells stained with annexin V-fluorescein isothiocyanate (FITC)/propidium iodide following treatment with different concentrations of Rapha Myr® for 24h. For the graph see main manuscript (Figure 8B)
